# Factors acting on *Mos1 *transposition efficiency

**DOI:** 10.1186/1471-2199-9-106

**Published:** 2008-11-26

**Authors:** Ludivine Sinzelle, Gwenhael Jégot, Benjamin Brillet, Florence Rouleux-Bonnin, Yves Bigot, Corinne Augé-Gouillou

**Affiliations:** 1 Université François Rabelais de Tours, GICC, UFR des Sciences & Techniques, Parc Grandmont, 37200 Tours, France; 2 CNRS, UMR6239, UFR des Sciences & Techniques, Parc Grandmont, 37200 Tours, France; 3 CHRU de Tours, UFR des Sciences & Techniques, Parc Grandmont, 37200 Tours, France

## Abstract

**Background:**

*Mariner-*like elements (*MLEs*) are widespread DNA transposons in animal genomes. Although *in vitro *transposition reactions require only the transposase, various factors depending on the host, the physico-chemical environment and the transposon sequence can interfere with the *MLEs *transposition *in vivo*.

**Results:**

The transposition of *Mos1*, first isolated from *drosophila mauritiana*, depends of both the nucleic acid sequence of the DNA stuffer (in terms of GC content), and its length. We provide the first *in vitro *experimental demonstration that MITEs of *MLE *origin, as small as 80 to 120-bp, are able to transpose. Excessive temperature down-regulates *Mos1 *transposition, yielding excision products unable to re-integrate. Finally, the super-helicity of the DNA transposon donor has a dramatic impact on the transposition efficiency.

**Conclusion:**

The study highlights how experimental conditions can bias interpretation of *mariner *excision frequency and quality. *In vitro*, the auto-integration pathway markedly limits transposition efficiency to new target sites, and this phenomenon may also limit events in the natural host. We propose a model for small transposons transposition that bypasses DNA bending constraints.

## Background

*Mariner-*like elements (*MLEs*) are class II transposons found in most eukaryotic genomes. These elements are discrete DNA fragments that are able to move around within eukaryotic genomes, and in some cases they make up a high proportion of these genomes. *MLEs *consist of a DNA fragment of 1200 to 2000 base pairs (bp) that contains a single transposase-encoding gene without an intron. This gene is flanked by two short inverted terminal repeats (ITRs) of 19 to 40 bp in length. The widespread occurrence of *MLEs *in animal genomes is considered to be attributable to the fact that their transposition does not require host factors since, *in vitro*, the transposase (Tpase) encoded by these elements is all that is required to catalyze all the transposition steps [1]. The requirements for *mariner *transposition seem to be very simple, involving just two ITRs separated by a DNA fragment, a Tpase source, and magnesium cations as cofactors [1-5]. However, several factors can impair the ability of *MLEs *to achieve their own transposition *in vivo*.

Physical and biochemical conditions may interfere with the transposition efficiency. It has previously been demonstrated that *in vitro *the *Himar1 *Tpase (HIMAR1) is about twice as efficient at 28°C as at 23 or 40°C [1]. In addition, data from *in vitro *and *in vivo *assays have shown that *MLE *nucleic acid components are also interfering factors. The quality of the ITR sequence is crucial for the transposition efficiency of *Mos1 *[3,6], and the untranslated regions (UTRs) located between the ITR and the Tpase ORF at both ends of the transposon act as transposition enhancers [7]. Inner regions of the transposon also modulate transposition efficiency *in vivo*, since transposition depends on the size of the DNA stuffer between the ITRs [4,8], and also in some cases to the quality of this inner sequence [9].

So far, studies investigating factors that modulate *Mos1 *transposition have been fragmentary [10-13]. *Mos1 *is a potential tool in gene transfer and mutagenesis, and so we think that it is important to investigate these factors in more detail. In this study, we investigated the impact on the ability of *Mos1 *to transpose of two factors: (1) the DNA configuration of the transposon donor, and (2) the properties of the stuffer.

Our main findings concern four points. First, we show that the nucleic acid sequence of the DNA stuffer (in terms of GC content), and its length, can markedly impair transposition efficiency. Second, our study provides the first *in vitro *experimental demonstration that MITEs of *MLE *origin are able to transpose. We therefore propose a model for the physical constraint associated with the size of these small transposons. Third, our studies show that excessive temperature down-regulates *Mos1 *transposition, yielding excision products that are unable to re-integrate. Finally, we have demonstrated that super-helicity of the transposon donor has a dramatic impact on transposition efficiency.

## Results

### Impact of the stuffer size on *Mos1 *transposition

It had already been pointed out that increasing the stuffer size alters the transposition efficiency for *Himar1 *[4] and *Mos1 *[8]. We re-investigated this feature for *Mos1 *using 3Tet3 transposons enlarged from the 3' end of the tetracycline marker in bacterial transposition assays. Our findings confirmed that increasing the stuffer size did indeed dramatically decrease the transposition efficiency (Table 1). The impact of decreasing stuffer size was also investigated using *Mos1 *pseudo-transposons made with small marker genes encoding resistance to zeocin, blasticidin, and puromycin (375-bp, 423-bp and 662-bp respectively). Our findings show that 3Bla3, 3Zeo3 and 3Puro3 transposons were able to transpose efficiently (Table 1).

**Table 1:** Transposition frequencies of pseudo-Mos1 with various lengths

**Pseudo-Mos1**	**Transgene size (bp)**	**Transposition frequencies**
3Zeo3	375	3.1 × 10^-4^
3Bla3	423	0.9 × 10^-4^
3Puro3	622	4 × 10^-4^
3Kana3	1185	0.9 × 10^-4^
3Tet3	1191	1 × 10^-4^
Enlarged 3Tet3	2500	1.2 × 10^-4^
	5000	5 × 10^-6^
	7000	3.6 × 10^-8^
	12500	<10^-9^

### Transposition of Minute transposon

To transpose, the *Mos1 *transposon has to be bent, bringing the ITR together [14]. DNA fragments that are longer than the bending persistence length (90-bp) are spontaneously bent, with little force. In contrast, significant bending of DNA fragments less than 90-bp in length requires considerable force [15]. These constraints should prevent the transposition of *MLE *with stuffer DNA less than 90-bp in length: this looks in contradiction with the presence of thousands of 80-bp *Hsmar1 MLE *interspersed within the human genome [16,17].

**Figure 1:**
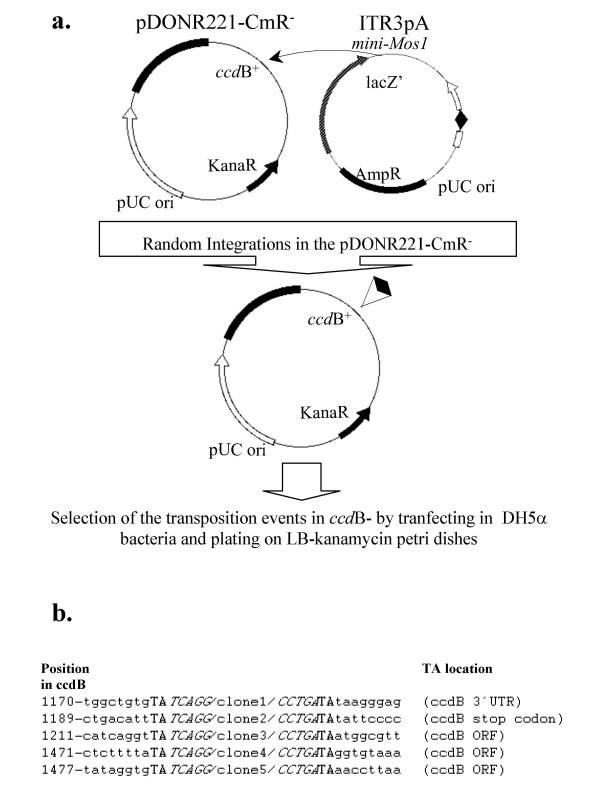
***In vitro *transposition of minute *Mos1 *transposons**. (a) Diagrammatic representation of the transposition assay. The minute *Mos1 *(mini-*Mos1*) is represented by a double arrow in black. ITR3pA3 and pDONR221-CmR^- ^were incubated for 1 hour with 80 nM of purified MOS1. The resulting plasmids were then transferred into DH5α *E.coli *and plated on kanamycin. Twenty clones were then sequenced, five of which corresponded to true transpositions. (b) Nucleic acid sequences of the five mini-*Mos1 *integration sites in the *ccd*B^- ^gene. Sequences were obtained from plasmids conferring a *ccd*B toxin survival. The TA dinucleotides duplicated at the target site are shown in bold, the outer ends of the ITR are in italics and the transposon sequence is indicated by"/cloneX/".

**Figure 2:**
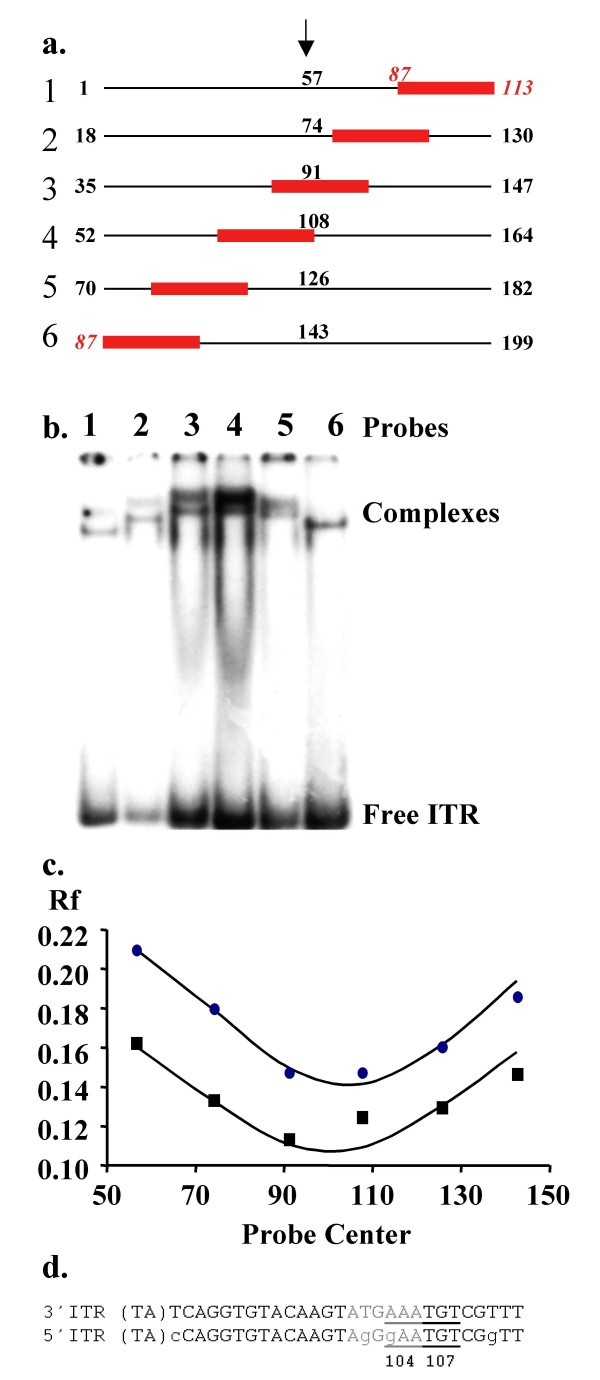
**Bending of the ITR by MOS1**. (a) DNA probes used in ITR bending experiments. The nucleotide positions of the 28-bp ITR (gray box) inside the DNA probes are 87 to 113. Six circular permuted fragments were used, which were equal in size (113 bp) and contain either the 3' or the 5'ITR. The arrowhead marks the center of each probe. The corresponding nucleotide positions are indicated. (b) EMSA with MOS1 and the six probes containing the 5'ITR. SEC1 and SEC2 are the lowest and the highest complexes respectively. (c) Mapping the bending center of the 5'ITR containing fragments. The relative mobility (Rf = migration distance of the MOS1-5'ITR complex/mobility distance of free DNA) of each probe was plotted as a function of the position of the center of each probe (shown in A). A sinusoid fit of the data points allowed us to map the center of the MOS1-induced bending. In this representative experiment, the center of the bending core is at position 105 (19^th ^bp in the 5'ITR sequence) for SEC1 (black points), and at position 102 (16^th ^bp in the 5'ITR sequence) for SEC2 (black squares). (d) Localization of the regions bent by MOS1 on the 3' and 5'ITR, for SEC2 (in gray) and SEC1 (underlined). The bending centers are indicated in upper-case letters. The sequence differences between the two ITRs are indicated in lower-case letters in the 5'ITR sequence.

Since no antibiotic marker less than 90-bp was available, we designed a novel transposition assay (Fig. 1a). This assay was monitored *in vitro*, ensuring that the bending of the DNA stuffer was not due to a host factor. We were able to recover surviving DH5α bacteria, indicating that the 122-bp *Mos1 *transposon was indeed able to transpose within the *ccd*B+ gene. Sequencing the *ccd*B+ gene indicated that five of the twenty clones analyzed corresponded to real transpositions of the 122-bp *Mos1 *transposon, as shown by the duplication of a TA dinucleotide at the integration site (Fig. 2b). The other clones are due to bacteria that naturally escape the toxin encoded by the *ccd*B+ gene, as indicated by the Manufacturer (see Materials and Methods).

These findings were not consistent with what we know about the bending ability of small, double-stranded DNA fragments. Since there was no helping host factor in our *in vitro *assay, we searched for sources of flexibility that would allow the bending required for assembling the synaptic complex.

The binding of a protein to DNA is often associated with a bend in the DNA. Bending of ITRs has been demonstrated for Tn*7*, Tn*5 *and IS*903 *bacterial transposons [18-20]. We therefore first investigated whether MOS1 bends its ITR. EMSA were performed using six 113 bp fragments containing the 28 bp 3'ITR or 5'ITR at various positions (Fig. 2a) and purified MBP-MOS1. The mobility differences between the MOS1-ITR complexes formed with the six different probes indicated that the MOS1 did bend the ITR. The possibility that these bends were due to single strand DNA cleavage at the outer or the inner extremity of the ITR was ruled out as all the experimental steps were carried out for 15 minutes at 4°C, conditions for which the cleavage activity of the Tpase is inhibited. Fig. 2b shows a representative data obtained with probes containing the 5'ITR. Similar data were obtained with probes containing the 3'ITR. The Rf of all the observed complexes was measured and plotted *versus *the position of the center of each probe (Fig. 2c). For each probe, two single end complexes (SEC1 and SEC2) were obtained, as expected [3]. The sinus curves calculated from our data allowed us to map the center of the MOS1-induced bend, located at the lowest point of the curve. This was at position 107 (± 3 bp) for SEC1, which located the bending core around the 21^st ^nucleotide of both ITR (Fig. 2d). For SEC2, the lowest point of the curve was around position 104 (± 3 bp), which located the bending core around the 18^th ^nucleotide for both ITRs (Fig. 2d). These values are the means (± SD) for at least five independent experiments. Zhou's equation [21] was used to determine bending angles of 89.7° (± 0.2) and 89.7° (± 1) for the SEC1 formed with the 3' ITR- and the 5' ITR-containing fragments respectively, and 89.2° (± 0.5) and 89.7° (± 1) for the SEC2 formed on the 3' ITR- and the 5' ITR-containing fragments respectively. In conclusion, our results indicated that both 3'- and 5'-ITR were bent at angles of about 90° as a result of MOS1 binding. Interestingly, these angles may facilitate the assembly of a transposition complex in a minute transposon, such as the-one investigated here, since the stuffer fragment needs only form an angle of less than 90° to bring the transposon ends together which is quite possible for a 66-bp DNA segment [15].

**Figure 3:**
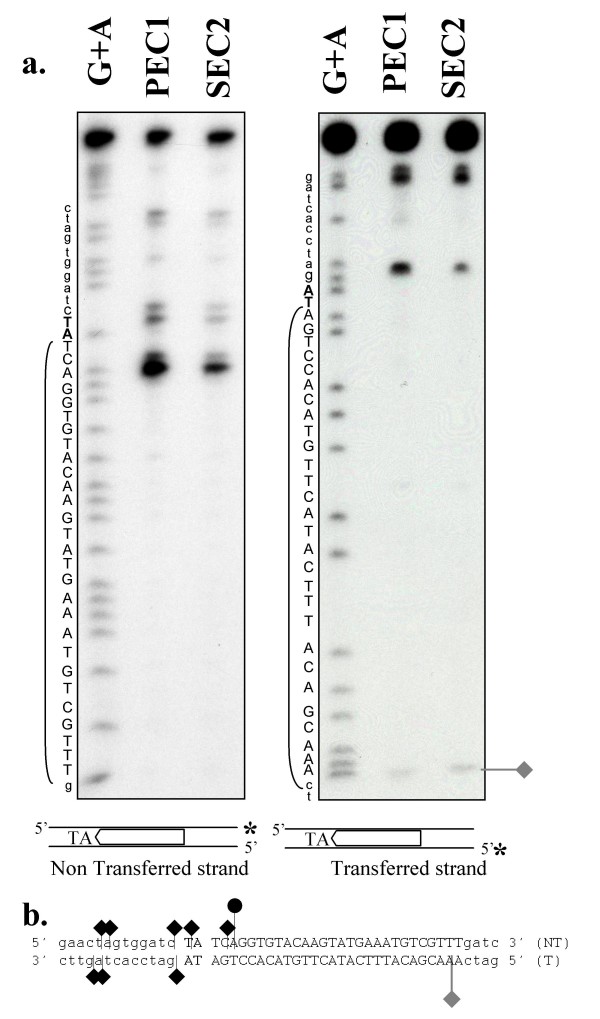
**Cleavage positions on preformed PEC1 and SEC2**. (a) The DNA contained in SEC2 and PEC1 was eluted from EMSA gel and analyzed on a sequencing gel. An asterisk marks the position of the ^32^P. The sequence reaction (G+A) of each probe was loaded as a marker, and the sequence was indicated in the left margin. Brackets indicate the location of the ITR region, the flanking TA dinucleotide is shown in bold, upper-case letters, and the non-ITR flanking DNA is shown in lower-case letters. (Left panel) Cleavage products obtained using ITR70α, labeled on the non-transferred strand as a probe. (Right panel) Cleavage products obtained using ITR70γ, labeled on the transferred strand as a probe. (b) Sequence of the DNA used in cleavage-site determination. The "minor" cleavage sites at the outer ITR extremity are indicated by black diamonds, and the major site by black circle, located either on the non-transferred strand (NT) or on the transferred strand (T). Cleavage located at the inner ITR extremity of the T strand is indicated by a gray diamond.

Another source of DNA flexibility, such as single strand DNA cleavage at the inner end of the ITR, was also investigated. The cleavages produced by MOS1 in SEC2 and PEC1 were therefore re-investigated, taking into account the inner part of the ITR (Fig. 3a). We have already published that the ITR is mainly cleaved at the 3'-end of the transferred strand and at positions +2 and +3 on the non-transferred strand, although several other cleaved positions were detected in the flanking DNA on both strands (Fig. 3b, black marks) [22]. Analysis of the bottom part of the gel also reveals that a single strand DNA cleavage occurred on the transferred strand at position 27, which is located at the inner end of the ITR (Fig. 3a, gray mark).

### Impact of stuffer sequence on *Mos1 *transposition

Two questions were addressed concerning the impact on transposition efficiency of the GC content of the transposon sequence, and of the number of TA dinucleotides in the transposon sequence.

**Figure 4:**
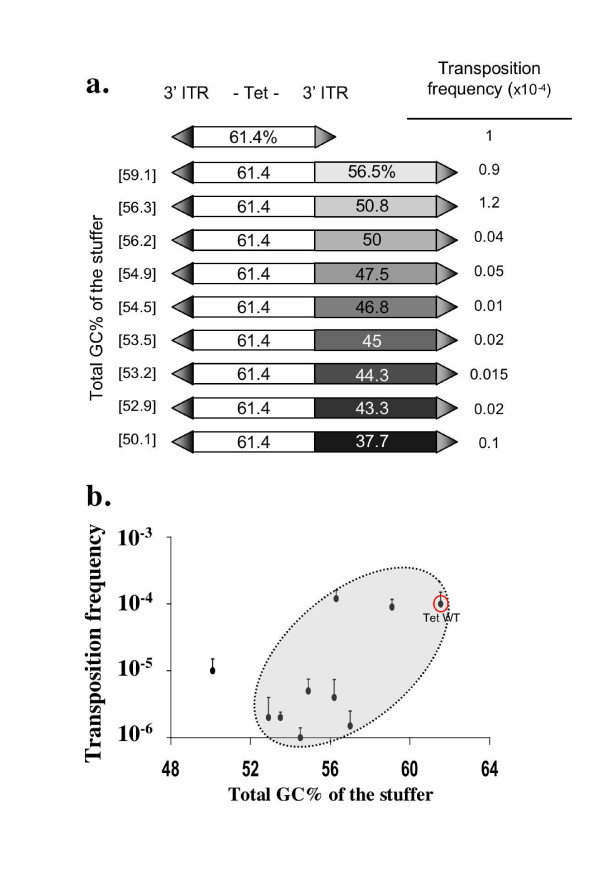
**Impact of the stuffer properties on *Mos1 *transposition efficiency**. Transposition assays in bacteria were performed using pKK-MOS1 as the Tpase source. (a) Transposition efficiency of Tet stuffer with various GC content. The Tet genes and the added fragments are shown as boxes; The GC contents of the Tet genes and/or added fragments are indicated in each box. ITRs are represented as arrows, flanking the stuffer. The total GC content of each stuffer is bracketed on the left. The transposition efficiency of each construct is indicated on the right. (b) Variation of transposition efficiency (vertical axis) with the GC content of stuffers (horizontal axis). The reference construct (3Tet3) is indicated. The axis of the ellipse follows the correlation between transposition frequencies and GC content of the stuffers. Only one construct (the last construct drawn in a.) does not fall within this ellipse. Averages and standard errors were calculated from at least five replicates.

We first prepared nine pseudo-*Mos1 *with GC contents ranging from 50.1 to 59.1%. The reference construct was the pBC-3Tet3, which has a GC content of 61.4%. In bacterial transposition assays, the transposition efficiency of the ten pseudo-transposons ranged over two orders of magnitude (Fig. 4a). The data were plotted, and the correlation between the two parameters (CG content *versus *transposition frequency) was tested using the Spearman correlation test with a significance threshold of 0.05 (XLSTAT2007). Nine of the 10 constructs plotted in this analysis were included in the ellipse (Fig. 4b), and the correlation between GC content and transposition frequency had a coefficient of about 0.8. This coefficient was significant enough to consider that there was a correlation between GC content and transposition frequency. However, the facts that one transposon construct fell outside the ellipse, and that two other transposon constructs with GC contents close to 56% differ by more than one order of magnitude indicates that GC content is not the only sequence parameter that affects the stuffer quality for transposition.

We then prepared two modified genes encoding for tetracycline resistance: the first was TA-rich (87 TA dinucleotides), and the second was TA-poor (14 TA dinucleotides), but this did not have any marked effect on the percentage of GC (58 and 62% respectively). These two genes were used as markers in transposition assays in bacteria. It was expected that reducing the TA density would result in increased transposition efficiency, simply because some suicide auto-integrations would be avoided. We observed no correlation between the TA numbers (or TA density), and the transposition frequency (Table 2). This point was confirmed by the fact that a bleomycin resistance gene without the TA dinucleotide (3Zeo3 [noTA]) gave the same result in transposition assays in bacteria as the wild type bleomycin resistance gene (3Zeo3; Table 2).

**Table 2:** Transposition frequencies of pseudo-Mos1 having various TA numbers

**Pseudo-*Mos1***	**TA density/100 bp (total TA number)**	**Transposition frequencies**
3Tet3	3.1 (37)	1 × 10^-4^
3Tet3 [TA-rich]	7.5 (87)	0.25 × 10^-4^
3Tet3 [TA-poor]	1.2 (14)	0.1 × 10^-4^
3Zeo3	0.2 (1)	3.1 × 10^-4^
3Zeo3 [noTA]	0 (0)	4 × 10^-4^

### Impact of temperature on *Mos1 *transposition efficiency

**Figure 5:**
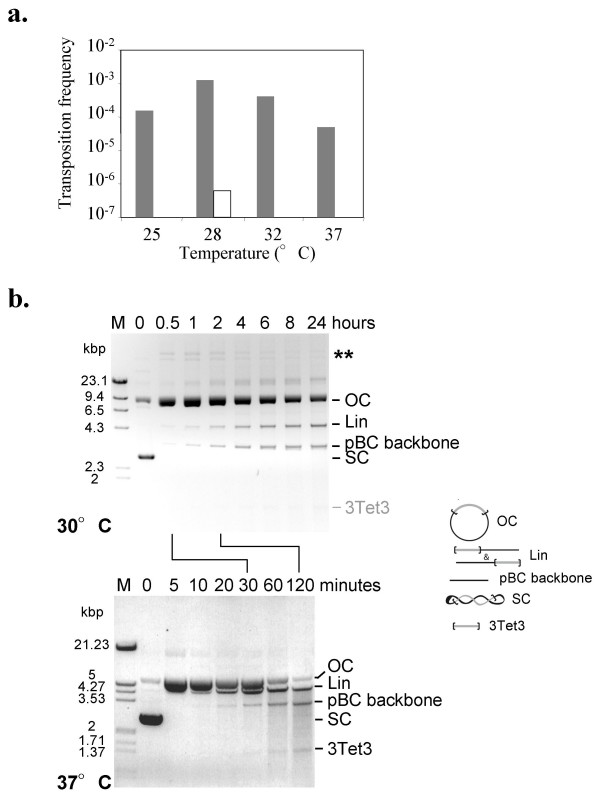
**Impact of the temperature on *Mos1 *transposition**. (a) Effect of the temperature (horizontal axis) on the transposition efficiency (vertical axis). *In vitro *assays were performed using two plasmids (pET-3Tet3 as the transposon donor, and pBC-SK+ as the target for integration) and purified MBP-MOS1. Two different shapes of pBC-3Tet3 were used: the super-helical form (gray bars) and that linearized with *Nco*I (empty bars). (b) Effect of temperature on the excision. Time course analyses were done using super coiled pBC-3Tet3 as pseudo-*Mos1 *and purified MBP-MOS1. The assays were performed at 30°C (top panel) or 37°C (bottom panel) and the resulting products were loaded onto BET-stained agarose gel. Molecular weight markers are indicated in the left margin. The various products are depicted on the right and their positions on the gel are indicated. OC: open circle; Lin: linear; SC: super coiled; 3Tet3: excised transposon. On the top panel (30°C) the expected position of 3Tet3 is pointed out, but the corresponding product is not detected. Asterisks indicate the integration products, determined according to [38] and personal data.

*In vitro *transposition assays were performed at four temperatures, 25, 28, 32 and 37°C (Fig. 5a). Our results are in agreement with those previously obtained for HIMAR1 [4], as we found an optimal temperature of about 28°C, with no significant differences found between 28°C and 30°C (not shown). Since we had previously shown that MOS1 binds to its ITR with similar efficiency regardless of temperature [22], we therefore checked whether a temperature effect was observed for *Mos1 *excision or not. Time course excision assays were performed either at 30 or 37°C, using pBC-3Tet3 plasmid as the transposon donor, and purified MBP-MOS1. Changing the excision temperature led to two main differences (Fig. 5b). The first was that, at 37°C, a marked DNA degradation occurred, leading to the loss of about 50% of the DNA two hours into the reaction, whereas no DNA loss was observed after 24 hours at 30°C. The second difference was that, despite the DNA degradation, the pseudo-*Mos1 *(3Tet3) accumulated at 37°C, whereas it was not detectable at 30°C. This is not very surprising because an accurately excised transposon tends to be reinserted rather than accumulating. Indeed, insertion products were seen in the assay performed at 30°C, but not in the assay performed at 37°C (Fig. 5b, bands pinpointed by asterisks). This was in total agreement with the fact that more efficient transposition was observed at 30°C than at 37°C. The DNA degradation and the 3Tet3 accumulation detected at 37°C might be due to a single phenomenon: at 37°C, MOS1 cleaves DNA not only at the end of the ITR but anywhere in the DNA. This loss of specificity may promote the production of excised 3Tet3 with extremities that are unable to integrate.

### Organization of excised pseudo-*Mos1 *produced at 37°C

**Figure 6:**
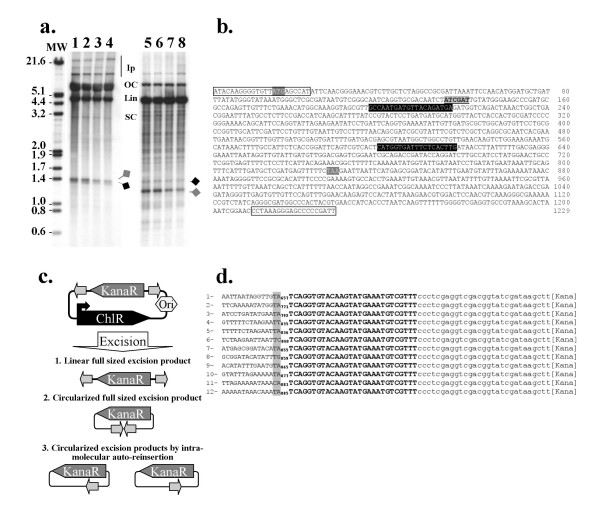
**Configuration of the excision products at 37°C**. (a) Southern blot analysis of excision products obtained by incubating pBC-3Kana3 with MOS1 at 37°C, for 15 (lanes 1 and 5), 30 (lanes 2 and 6), 60 (lanes 3 and 7), and 120 minutes (lanes 4 and 8). After deproteinizing, DNA samples were digested (lanes 5 to 8) by *ClaI*. Products were finally loaded onto 0.8% agar gel (20 × 24 cm), separated by electrophoresis, and blotted onto Nylon membrane. Hybridization was done with a P^32^-labelled fragment containing the kanamycin resistance gene. Ip: integration products, OC: open circle, Lin: linear; SC: super-coiled. Molecular weights (MW) are indicated in the left margin. Grey diamond: linear excised 3Kana3. Black diamond: circular excised 3Kana3. (b) Nucleic acid sequence of the kanamycin resistance gene cloned within the pBC-3Kana3. The motifs used to design the KanaSal1 and KanaHind3 primers are boxed at both ends of the sequence (Table 3). Motifs highlighted in black with the sequence typed in white were used to design the primers KanaJunc1 and KanJunc2 (Table 3). The start and stop codons of the kanamycin resistance gene are highlighted in dark gray and typed in white. The *Cla*I site is typed in black and boxed in gray. (c) Diagrammatic representation of the excision products obtained after incubation of pBC-3Kana3 with MOS1 at 37°C. Three forms of the excised transposon are drawn: linear (1) and circular (2) molecules containing a full-length transposon, and (3) circular molecules containing a truncated transposon, due to intra-molecular auto-integration after excision. ITR are represented by light gray arrows and promoters by 90°-angled arrows. KanaR: Kanamycin resistance gene. ChlR: CAT gene. (d) Sequences of the twelve excision products analyzed. The 3'ITR is in bold uppercase letters. On the right, the pBC MCS is in lowercase letters. On the left, the dinucleotide of the auto-integration site is in gray-boxed uppercase letters. Numbers flanking the insertion sites correspond to the position of insertion in the 3Kana3 pseudo-*Mos1*. They are the same as in (b). The sequence in 5' of the insertion site corresponds to the inner sequence for 3Kana3.

The organization of the pseudo-*Mos1 *that accumulated at 37°C was analyzed using a variant of the pBC3Tet3, pBC-3Kana3, a transposon donor with a unique *Cla*I site in 3Kana3 (Fig. 6a). In Southern blot analyses, pBC-3Kana3 is detected both as open circle and linear molecules, whereas the excised 3Kana3 accumulates as a double-band of 1.3 kbp (Fig. 6a). Due to the sensibility of the technique, integration events are also detected. The detection of a double-band of about 1.3-kpb (Fig. 6a, lanes 1 to 4, bands located by gray and black diamonds) suggests that excision products could exist both in linear and circular configurations. Southern analysis of the DNA samples digested by *Cla*I was then monitored. Once cleaved by *Cla*I, the band with the highest apparent molecular weight in undigested DNA samples was shortened accordingly to the position of the restriction *Cla*I site (Fig. 6a, lanes 5 to 8, bands located by a gray diamond). This suggests that this band corresponds to a linear excision product. In contrast, the band of lower intensity in undigested DNA samples is unaffected or only slightly by *Cla*I digestion, thus suggesting that it corresponds to a circular excision product or that it had no *Cla*I site.

**Table 3:** Oligonucleotides used in the study

***Primer Name***	Nucleic acid sequence
End modification of the kanamycin resistance gene	
KanaSal1	5'-AGCGTCGCATACAAGGGGTGTTATGAGCCAT-3'
KanaHind3	5'-ACCCAAGCTTAATCGGGGGCTCCCTTTAGG-3'
	
3Kana3 junction from the kanamycin resistance gene	
KanaJunc1	5'-TCATCTGTATAACATCATTGGC-3'
KanaJunc2	5'-CATGGTGATTTCTCACTTG-3'
	
*Stuffer fragments amplified from DpAV4 sequence referenced as AJ279812*	
3primeXba1	5'-GCTCTAGACGGACGTCCAATACATGATG-3'
5primeXba1-F1	5'-GCTCTAGACCGAAAAGATAGACAGTGTG-3'
5primeXba1-F2	5'-GCTCTAGAAACGTGACGCACATGGCTAT-3'
5primeXba1-F3	5'-GCTCTAGAAAACATCCTTCGCCCTGAAC-3'
5primeXba1-F4	5'-GCTCTAGAAGGATTGGTGGGATTTTCCG-3'
5primeXba1-F5	5'-GCTCTAGAACGGCAACTTTCGGAACTAC-3'
Test-orient-F	5'-GCGAATTGGCCCCTAGATTT-3'
	
	*Insertion site analyses in the ccdB gene*
ccdBup	5'-AGTCGTTCGGCTTCATCTGG-3'
ccdBdown	5'-ATCAGGAAGGGATGGCTGAG-3'
	
	*ITR cloning for circular permutation*
5'ITRup	5'-AGCTCGTTTACCAGGTGTACAAGTAGGGAATGTCGGTTCCC-3'
5'ITRdw	5'-GGGAACCGACATTCCCTACTTGTACACCTGGTAAACGAGCT-3'
3'ITRup	5'-AGCTCGTTTATCAGGTGTACAAGTATGAAATGTCGTTTCCC-3'
3'ITRdw	5'-GGGAAACGACATTTCATACTTGTACACCTGATAAACGAGCT-3'
	
	*Fragments for circular permutation*
O1	5'-GGGTTTTCCCAGTCACG-3'
O1R	5'-AACGACATTTCATACTTGTA-3'
O2	5'-ACGTTGTAAAACGACGGC-3'
O2R	5'-CTAGAGCGGCCGCGG-3'
O3	5'-CCAGTGAGCGCGCGTA-3'
O3R	5'-CGGGGGATCCACTAGTT-3'
O4	5'-TACGACTCACTATAGGGC-3'
O4R	5'-ATCGAATTCCTGCAGCCC-3'
O5	5'-CGAATTGGAGCTCGTTTAT-3
O5R	5'-GTATCGATAAGCTTGATATC-3'
O6	5'-ATCAGGTGTACAAGTATGAA-3'
O6R	5'-TTCCCTCGAGGTCGACG-3'

The excision products were therefore purified by agarose gel elution from untreated DNA samples. They were submitted to PCR using primers anchored in an inverse orientation in the sequence of the kanamycin resistance gene, KanaJunc1 and KanaJunc2 (Table 3 and Fig. 6b), in order to amplify ITR junctions looking like those isolated from *Bombyx mori *cells transformed by *Mos1*-based vectors [24]. The amplification of a fragment of about 950-bp is expected if the circular forms correspond to the circularization of an excised full-length transposon (Fig. 6c). Fragments ranging from 600 to 800-bp were obtained and cloned. Twelve were sequenced: they all contained the *Cla*I site, and a single ITR, inserted into or close to the kanamycin resistance gene (Fig. 6d). This fact, combined with the fact that they were shorter than expected, indicates that these fragments resulted from intra-molecular integration events. Among the twelve fragments analyzed here, four came from auto-integration events occurring in a dinucleotide that was not a TA but TT (sequence n°4), TC (sequence n°6), TG (sequence n°8) or CA (sequence n°11) dinucleotides.

In conclusion, our data are consistent with the fact that some of *Mos1 *excision products formed at 37°C are not able to perform integration into a target DNA. This is due to the formation of (1) linear products with inefficient extremities and (2) circular products resulting from intra-molecular auto-integration. Taken together, these events account for the decreased transposition efficiency observed at 37°C.

### Impact of the transposon donor configuration on *Mos1 *transposition

*In vitro *transpositions were performed using a linear plasmid donor of transposon (Fig. 5a, empty boxes) and compared to those obtained using a super coiled transposon donor (Fig. 5a, gray bars). Linearization of the transposon source dramatically reduced the transposition efficiency (more than 1000-folds) at 28°C, and hindered transposition at other temperatures.

**Figure 7:**
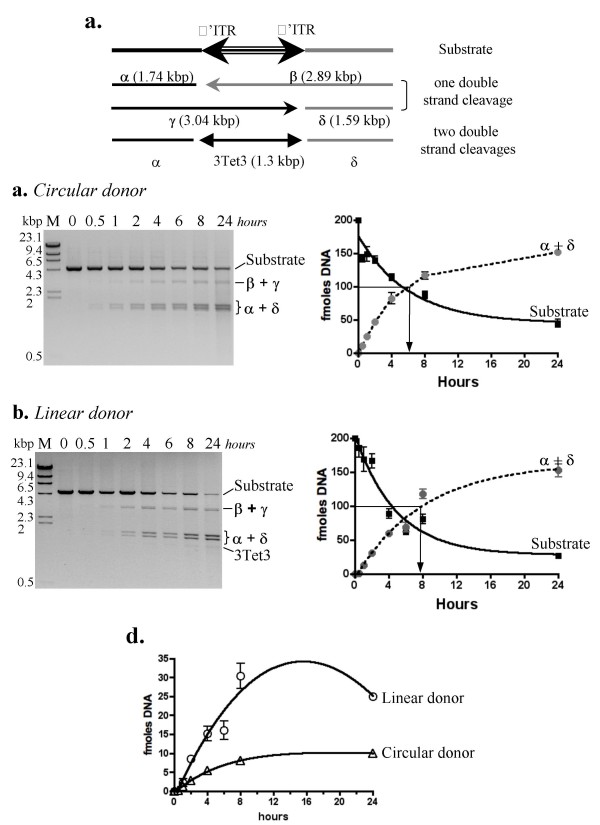
**Excision Time-course analysis at 30°C: circular *versus *linear**. (a) Diagrammatic representation of excision products obtained (after MOS1 activity) from an *Nco*I-linearized pBC-3Tet3: double strand DNA cleavages at one ITR yield two fragments (α and β) or (γ and δ). Double-stranded DNA cleavages at each ITR end yield three fragments (β, δ and 3Tet3). (b) Analysis of cleavage products obtained from super coiled pBC-3Tet3 DNA substrate. Reactions were performed from 0 to 24 hours at 30°C, using 80 nM MOS1. After deproteinizing, products were *Nco*I digested and loaded onto agarose gel (left panel). Molecular weights (in kbp) are shown on the left. The different DNA species observed are indicated on the right, using the same codification as in (a). After quantification (ImageGauge V4.22 software), DNA substrate and α + δ bands (in fmoles) were plotted as a function of time (right panel, Prism Software). Averages and standard errors were calculated from five independent replicates, using Prism software. T1/2 is the time required producing 50% of α + δ (in fmoles, indicated by an arrow). (c) Analysis of cleavage products obtained from a linear pBC-3Tet3 DNA substrate. Reactions were performed and analyzed as in (b). The only difference was that cleavage products were directly loaded onto agarose gel. (d) After quantification (ImageGauge V4.22 software), β + γ bands obtained in (b: circular donor) and (c: linear donor) were plotted (in fmoles) as a function of time (right panel, Prism Software). Averages and standard errors were calculated from five independent replicates (Prism software).

Excision efficiencies of a super-helical donor were then compared to those obtained with the same donor linearized by *Nco*I. When the super-helical pBC-3Tet3 was used as the substrate, products were digested by *Nco*I at the end of the experiment, making it possible to carry out direct comparisons. Excision assays have been first analyzed by tracing the dead-end products of the reaction, *i.e*. bands α and δ(Fig. 7a). Our data therefore show no major difference between the two reactions. Analyses of the time taken to obtain 50% of α and δ fragments (T1/2) indicated that it was about 6 and 8 hours for the super-coiled and linear donors, respectively (Fig. 7b and 7c, black arrows). Furthermore, the curves differ in shape, the production of α + δ (for the first points of time, *i.e*. the initial reaction speed) being faster with the circular substrate. This suggested differences between the two excision reactions.

This difference was studied measuring the patterns of appearance of the β and γ fragments that are not dead-end products of the reaction. Indeed, when *Mos1 *excision occurs within the synaptic complex, β and γ are not expected to accumulate, because cleavage is believed to occur in a concerted fashion at both ITRs. Much more β and γ accumulated when the donor was linear, rising to a peak before decreasing, as the result of cleaving the second ITR (Fig. 7d). In contrast, little β and γ accumulated when the substrate was super coiled, reaching a maximum of 10-fmoles. These data supported the idea that the concerted cleavage at both ITRs is reduced or abolished when the substrate is linear, a phenomenon that may account for an overall decrease in the transposition efficiency. Finally, transposition assays presented in Fig. 5a corresponded to 30 minutes reactions. At this time, excision and re-integration of the transposon from the circular substrate had already occurred (Fig. 7b, lane 0.5) whereas this had not occurred for the linear substrate (Fig. 7c, lane 0.5).

Data in this section indicates that the excision is probably one of the limiting factors that accounts for the drastic decrease in transposition efficiency when the donor is linear. At least two non-mutually exclusive explanations can be proposed: (1) excision from a linear donor is slower and (2) excision from a linear donor takes place independently at both ITRs (non-concerted excision).

## Discussion

In addition to requiring ITR and the Tpase, previous studies have demonstrated that the transposition of *MLE *or *TLE *requires critical parameters that interfere with transposition efficiency. These parameters include (1) Tpase concentrations, demonstrated *in vitro *for HIMAR1 [4] and MOS1 [7] and *in vivo *for MOS1 [25], (2) temperature [4], (3) size of the stuffer, demonstrated *in vitro *for *Tc1 *[26], (4) sequence of the stuffer, demonstrated *in vivo *for MOS1 [9], (5) structural configuration of the target DNA, demonstrated *in vitro *for *Tc1 *[27].

In this study, we have used an *in vitro *approach to address several parameters affecting *Mos1 *transposition.

### Parameters for optimal *mariner *transposition

Our results highlight the importance of the stuffer sequence since they show that the GC content is an important factor, even if it is probably not the only parameter that affects the stuffer performance for transposition. *In silicio *analyses  failed to establish a correlation between DNA curvature and/or flexibility and transposition efficiency.

Our results also confirm the impact of stuffer length on transposition efficiency, as already shown [4,8,28]. Length dependence of *Mos1 *transposition might rely on auto-integration after excision. This is supported by the fact that large sophisticated transposons such as the phage *Mu *or Tn*7 *have specific mechanisms that prevent the transposon from inserting into itself [29,30]. In contrast, simple elements such as *mariners *lack this function.

A third parameter that controls *Mos1 *transposition rate is the temperature. Interestingly, elevated temperature (37°C) promotes auto-integration events. Auto-integration does not absolutely require a TA dinucleotide. This fact, associated with the fact that the percentage of TA dinucleotides does not influence *Mos1 *transposition efficiency, rules out the possibility of designing large transgenes containing no (or only a few) TA to limit the length-dependence of *Mos1 *transposition.

### Mariner excision: making a molecule that produces efficient integration

**Figure 8:**
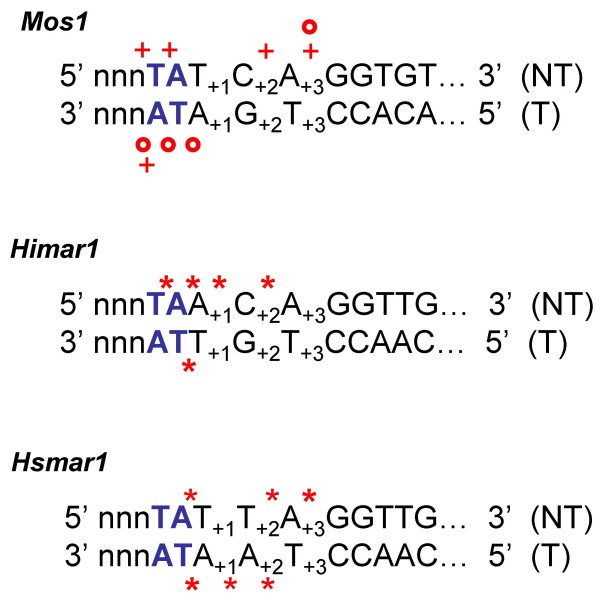
**Published data on *mariner *double stranded cleavages**. Data about cleavages *in vitro*. (T) is the transferred strand, and (NT) the nontransferred strand. Upper-case letters indicate the terminal DNA sequence at the right end of *Mos1*, and of both ends of *Himar1*, and *Hsmar1 *respectively. Lower-case letters indicate the flanking DNA. The TA dinucleotide flanking the element is in bold. Numbers indicate base pair positions relative to the transposon-donor junction. Crosses correspond to the cleaved positions mentioned in [22]; Circles correspond to the cleaved positions mentioned in [23]; Stars correspond to the cleaved positions mentioned in [1] and [17] for *Himar1 *and *Hsmar1 *respectively.

Data presented here highlight an important, although not new, idea: transposition efficiency is in great part controlled by the quality of the excision product. This is in contradiction with published data [1,17,22,23] that support the idea that cleavages at *MLEs *ends are far from accurate (Fig. 8). However, all these data were collected *in vitro*, using linear, double-stranded oligonucleotides. On the other hand, *MLE in vivo *excision footprints are often less variable, and closely match what the model predicts, *i.e*. hydrolysis at the 3' end of the element, and 5' break two or three nucleotides within the transposon [17,24,31]. This suggests that experimental conditions can bias the description of excision product ends.

Our data allow proposing that there is a relationship between the super-helicity of the donor DNA, and the production of efficient excision products, through the assembly of the synaptic complexes. Indeed, *Mos1 *excision from a super-coiled donor yields transposition that is 1000-fold more efficient than that resulting from a linear donor plasmid (10^-3 ^and 5 × 10^-7 ^respectively). These findings are fully consistent with super-helicity acting as a regulatory factor of *mariner *transposition *in vitro*, and probably *in vivo*. From this standpoint, *mariner *looks like many other transposons. For instance, Mu transposition requires DNA super coiling in the donor DNA [24] whereas Tn*10 *needs either DNA super coiling in the DNA substrate, or IHF acting as a "super coiling relief factor" [32]. Super coiling is probably not required for the chemical steps of the transposition, but might be a limiting factor for the assembly of *mariner *transposition complexes. Although *mariner *transposition has been shown to take place *in vitro*, we cannot rule out the possibility that host factors may assist the transposase during *in vivo *transposition.

### MITE transposition

**Figure 9:**
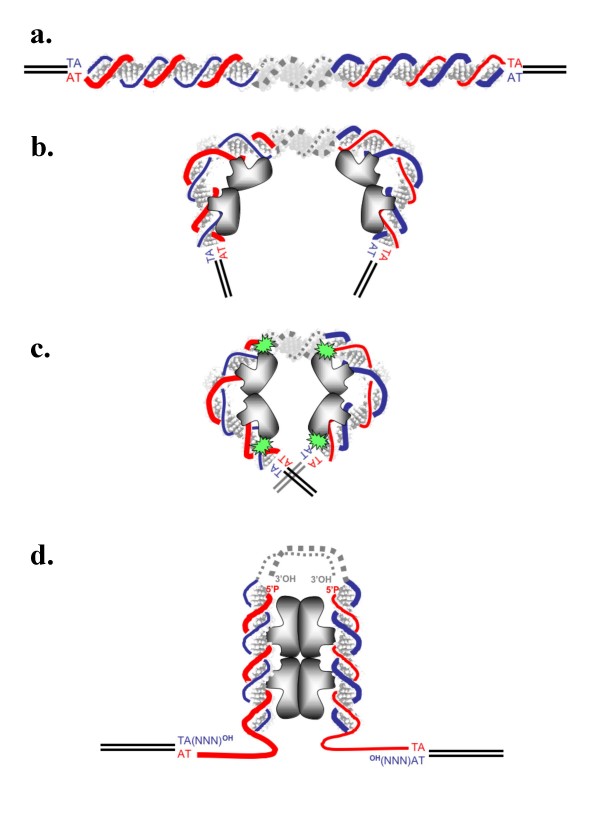
**Excision model for minute-*Mos1***. (a) Minute transposon structure: the positive DNA strand is indicated by a thin line, and the negative one by a bold line. The transferred and non-transferred strands are represented by red and blue lines respectively, on each inverted repeat. The duplicated TA dinucleotide at the insertion site was located on both strands. (b) Binding of one MOS1 dimer to each ITR bends it at an angle of about 90°. (c) In agreement with the data reported here, cleavages occurred at the inner extremities of both ITRs on the transferred strands. In accordance with previously published data, cleavages occurred at the outer extremities of the ITR, on the non-transferred strands. (d) The single strand nicks at both inner extremities of the ITR allocated flexibility to both non-nicked strands, thus allowing 180° flipping, and the PEC2 assembly. Full excision of the transposon occurred after the second cleavages at the extremity of both ITRs.

MITEs are small, repetitive, DNA elements ranging in size from approximately 80 to 500 bp that are interspersed within eukaryotic genomes. Their sequence is palindromic, AT-rich, and has no coding capacity. For their mobility, they generally use a source of Tpase *in trans*, originating from a related complete transposon copy [33]. Our data indicate that the size of MITEs of *MLE *origin does not limit their ability to transpose. For the smallest ones, found in the human genome (80-bp) or in that of the ant *Messor bouvieri *(130-bp, [34]), the binding of MOS1 causes major ITRs bending, thus bringing the two ends of the transposon close to each other. In addition, the inner ITR cleavage by MOS1 could also allows the ITR bending. Similar cleavage was found on the non-transferred strand of the *Hsmar1 *ITR cleaved by HSMAR1. No host factor is therefore required to bend the DNA and assemble the synaptic complex (Fig. 9).

This model provides a very satisfying explanation of the mobility of the *MLE *MITEs, because it explains how a minute *Mos1 *can assemble a functional synaptic complex similar to that of the full-length *MLE*. Moreover, it is supported by *in vitro *transposition data. However, it does not explain why most of the MITEs, including those derived from *MLE*, display a palindromic organization throughout their sequence. In contrast to what was previously proposed [35], our data suggest that complete palindromy is not a requirement for MITE mobility, and that it could therefore result from other constraints.

## Conclusion

Non-viral vectors are presently invading the world of gene transfer and mutagenesis. Among the tools that are currently under evaluation, the transposon *Mos1 *raises a growing interest. In fact, it is perceived as a small versatile and quasi-universal means of transforming a variety of cells, except mammal ones [13]. However, as for any new tool, it is necessary to control every parameter driving its transposition, and, in a first place, the factors that might limit its efficiency and specificity for DNA transfer. In the present work, we have investigated several parameters, particularly those related to the structure of the transposon.

In this respect, we have demonstrated that the DNA sequence (in term of GC content) as well as the length of the inner part of the transposon have a crucial effect on the transposition efficiency. Our results provide, for the first time, an experimental *in vitro *demonstration that MITEs of *MLE *origin that are as small as 80–120 bp are able to transpose. Hence, we propose a model that bypasses the physical constraints associated to the transposition of such small transposons. We have also demonstrated that the super-helicity of the transposon impacts considerably on the efficiency of transposition. Finally, we have found that *mariner *transposition is dependent on the temperature. The fact that higher temperature disfavors re-integration of the excision products might be related to the transposase enzymatic properties.

## Methods

### DNA manipulation

A double-stranded 3'ITR oligonucleotide with *Bam*HI extremities was cloned at the *Bam*HI site of pBS II SK+ (Stratagene), to produce pBS-3'ITR. This plasmid was subjected to *Eco*RI/*Xba*I cleavage, yielding the ITR70 fragment (*i.e*. a fragment 70 bp in length) that was then agarose-purified. After precipitation, the DNA concentration was estimated on BET-stained agarose gel.

Analysis of the cleavages occurring in SEC2 and PEC1 was performed using ITR70 labeled on one strand. To obtain single-strand labeling of the non-transferred strand (giving ITR70α), the ITR70 fragment was filled-in at the *Eco*RI site using the DNA polymerase Klenow fragment and dATP α-^32^P without dCGT. To obtain single-strand labeling on the transferred strand (giving ITR70γ) the pBS-3'ITR was digested by *Xba*I and dephosphorylated using CIP (Promega) before *Eco*RI digestion. After purification, the *Xba*I/*Eco*RI fragment was labeled at the dephosphorylated end using the T4 polynucleotide kinase (Promega) and ATP γ-^32^P.

### Polymerase chain reaction

The sequences and properties of the oligonucleotides used for the PCR amplification involved in plasmid constructs are shown in Table 3.

PCR amplifications of fragments smaller than 2 kbp were performed on 1 ng DNA. PCR reactions were done in 10 mM Tris-HCl, pH 9, 1.5 mM MgCl_2_, 50 mM KCl, and 0.1% Triton X100, 150 mM of each dNTP, and 0.1 mM of each oligonucleotide in a 50 μl reaction volume containing 1 unit of Taq DNA Polymerase (Promega). Each PCR was carried out in a programmable temperature controller (Eppendorf) for 30 cycles. The cycle was as follows: denaturing at 94°C for 1 min, annealing at 55°C for 30 sec, and extension at 72°C for 1 min. At the end of the 30^th ^cycle, the heat-denaturing step was omitted, and extension was allowed to proceed at 72°C for 7 min.

Fragments larger than 2 kbp were amplified using the Expand™ Long Template PCR System, under the conditions specified by the supplier (Roche Molecular Biochemicals). Briefly, reactions were done on 10 ng of gDNA in 1× buffer 1 or 2, 350 μM of each dNTP, 0.05 nM of each oligonucleotide in a 50-μl reaction volume, with 3.5 units of Taq/Tgo DNA polymerase mixture. Each PCR was carried out for 30 cycles comprising denaturing at 94°C for 30 sec, annealing at 61°C for 1 min, and extension at 68°C for 10 min. At the end of the 30^th ^cycle, the heat denaturing step was omitted, and extension was allowed to proceed at 68°C for 15 min.

### Proteins

The pMAL-c2-MOS1 encoding the wild type MOS1 (amino acids 1 to 345) was used as previously described, and the Tpase was produced and purified as a fusion protein linked to maltose-binding protein (MBP) [2]. MBP-MOS1 was used instead of MOS1, because although it has the same specific activity it is much more stable during purification, biochemical assays and *in-vitro *transposition assays. Henceforth in the text, the terms "transposase", "Tpase" and "Tpase molecule" all refer to a single subunit of MOS1.

The amount of MOS1 present after purification was determined on SDS-PAGE. Various amount of the samples for analysis were loaded onto the gel, and co-migrated with a range of dilutions of BSA. After staining with colloidal Coomassie dye, the gels were scanned, and the final concentrations were determined using the Molecular Analyst software (Biorad, [36]).

### Plasmids for transposition assays

#### Tpase source

A strong, IPTG-dependent MOS1 expression vector was constructed in the pKK-233 plasmid (Clontech) to obtain pKK-MOS1 [3]. This plasmid was used for transposition assays in bacteria.

#### Transposon donor

A pseudo *Mos1 *donor plasmid was constructed with two 3'ITRs flanking the promoterless tetracycline resistance gene of pBR322 (Tet), as previously described [3], and designated pBC-3Tet3.

Several variants of pBC-3Tet3 were prepared: pET-3Tet3 was made by cloning 3Tet3 transposon, between the *Sac*I-*Bss*HII sites of the pET26b(+) (Novagen). pBC3Kana3, which contains a promoterless gene encoding the kanamycin resistance instead of the tetracycline gene. Three plasmids, lacking the promoter of one of the genes encoding antibiotic resistance, were designated pBC-3Bla3, pBC-3Zeo3 and pBC-3Puro3. They contained the blasticidin resistance gene of pORF39-Bsr (InvivoGen), the bleomycin resistance gene of pORF39-Sh-ble (InvivoGen) and the puromycin resistance gene of pORF39-Pac (InvivoGen) respectively. A pCR-Script Amp plasmid containing a minute pseudo-*Mos1 *transposon of 122-bp, named ITR3pA3 [see Additional file 1], was also prepared.

To assay DNA stuffers larger than 1.2-kbp, four fragments of about 1.3-, 3.8-, 6.3- and 11.3-kbp were amplified by PCR from a 12255-bp segment contained in the Ascovirus DpAV4a (Acc N°AJ279813). Each fragment was inserted at the *Xba*I site of the pBC-3Tet3 located just after the stop codon of the tetracycline resistance gene. These constructs contained DNA stuffers of 2.5-, 5-, 7.5- and 12.5-kbp respectively.

To determine the impact of the sequence quality of the DNA stuffer, nine variants of pBC-3Tet3 were constructed by inserting into each of them amplified (primers in Table 3) 1.1 kbp fragments with differing GC contents that corresponded to untranscribed sequences in bacteria [see Additional file 1]. These constructs were designated pBC-3Tet3-F1, -F6, -F46, -F108, -F116, -F193, F240, F289, and F523 respectively.

Three other variants were constructed. The first, corresponding to the bleomycin resistance gene without the TA dinucleotide (pBC-3Zeo3 [noTA]), was synthesized using the Quick Change^® ^Site-Directed Mutagenesis Kit (Stratagene), using the pBC-3Zeo3 as the template, and contained an Y75F substitution in the bleomycin resistance gene, that did not alter the activity of the protein. The other two contained tetracycline resistance genes with either a high dinucleotide TA content (87 TA), or a low dinucleotide TA content (14 TA) that differ from that of the wild type version of the tetracycline resistance gene (37 TA), but without any change in the encoded protein sequence. These two tetracycline genes were synthesized (ATG bio Synthetics) and subcloned in the pBC-3-3, to yield two constructs designated pBC-3Tet3 [TArich] and pBC-3Tet3 [TApoor] respectively. After sequencing, the three resulting plasmids were used in bacterial transposition assays.

### Transposition assays in bacteria

JM109 competent cells were freshly co-transformed with pKK-MOS1 and one of the pseudo-*Mos1 *donors, and plated on LB-agar containing ampicillin (100 μg/ml) and chloramphenicol (150 μg/ml). Donor plasmids with a pBC backbone were also used as integration targets, since the CAT gene acts as a strong integration hot spot for *Mos1 *[3]. Four to six colonies containing both plasmids were grown overnight at 37°C in 2.5 ml LB medium containing ampicillin and chloramphenicol. 2.5 ml of fresh LB medium containing ampicillin and chloramphenicol was inoculated with 250 μl of the overnight culture. The cells were grown for 1 h at 37°C, and transposition was induced with 1 mM IPTG. The cells were grown for 5 h at 32°C (except in experiments investigating the impact of temperature), and transpositions were detected by plating the cells on LB medium containing tetracycline (20 μg/ml), kanamycin (100 μg/ml), blasticidin (100 μg/ml), zeocin (25 μg/ml) or puromycin (250 μg/ml), depending on the donor used in the assay. Cells were titrated by plating an appropriate dilution of the cultured cells on LB medium. The transposition frequency was calculated as the number of antibiotic-resistant cells divided by the total number of cells [3]. This ratio corresponds to a number of events per cell. In tests with very low transposition frequencies, the cells were grown for 20 h (instead of 5 h) at 32°C, and then transferred to 25 ml fresh antibiotic-free LB medium. The cells were then grown for another 6 hours at 32°C before detecting the transposition events as described above.

### *In vitro *transposition assays

The procedure was based on that described in [1]. Three assays were performed.

The first involved homo-plasmid transposition: 20 μl-reactions were carried out in 10 mM Tris-HCl [pH9], 50 mM NaCl, 0.5 mM DTT, 20 mM MgCl_2_, 0.5 mM EDTA, 100 ng BSA, 80 nM MBP-MOS1 to which 600 ng of pBC-3Tet3 had been added for 30 min at 30°C. The second involved hetero-plasmid transposition, and was carried out under similar conditions using 300 ng of pET-3Tet3 as the transposon donor (linear or super coiled), and 300 ng of pBC-SK+ (Stratagene) as the integration target. Linear donors were prepared from pET-3Tet3 digested by *Nco*I.

Reactions were stopped by adding 10 μl of stop solution (0.4% SDS, 0.4 μg/ml proteinase K) and incubated for 30 min at 37°C. Reactions were then phenol-chloroform extracted, and nucleic acids were ethanol precipitated using 1 μg/μl of yeast tRNA as carrier. The precipitated reaction products were resuspended in 10 μl of sterile water. 2 μl of reaction products, supplemented with 0.01 ng of pBS SK+, were used to transform 45 μl of competent *Escherichia coli *by electroporation (2 mm cuvette, 5 ms, 1.5 kV in a MicroPulser™ Bio-Rad). Cells were grown at 37°C for 1 hour and aliquots were plated on LB-ampicillin (to monitor the transformation efficiency), LB-tetracycline and LB-chloramphenicol (to monitor transposition rates = clone number on LB-tetracycline/clone number on LB- chloramphenicol).

The third assay was also a hetero-plasmid transposition assay (with similar incubation conditions as before), but used 300 ng of ITR3pA3 as the minute *Mos1 *donor, and 300 ng of pDONR221-CmR^- ^as a target for integration. This 3599-bp plasmid contained the ccdB gene that encoded a toxin lethal for the DH5α strain *of E. coli*, making it possible to select integration events. It was made from pDONR221 (Invitrogen) by removing the *Bam*HI-*Eco*RV fragment, containing the *cat *gene and located between positions 1833 and 3000, in order to avoid integration interference. As indicated by the manufacturer, using pDONR221 yields a background of about 10^-4 ^naturally resistant DH5α. Insertion sites of the pseudo *Mos1 *were amplified by PCR, using primer ccdBup and ccdBdown (Table 3), cloned in pGEM-T Easy (Promega), and sequenced by MWG biotech (Germany).

### Excision assays

Excision assays were performed in the same conditions than *in vitro *transposition assays. Reactions were performed using 600 ng of a single transposon donor (pBC-3Tet3 or pBC-3Kana3), either super coiled or linear and at different temperatures, as indicated in the text. At the end of the reaction, products were directly loaded on 0.8% BET-agarose in 1× TBE buffer.

Southern blots, and hybridizations were performed according to standard procedures [37]. After electrophoresis, samples were blotted on nylon membranes ((NylonN^+^, ICN products) and hybridized with a *Cla*I-*Hin*dIII fragment purified from pBC-3Kana3 and containing the promoterless gene encoding kanamycin resistance. This probe was randomly labeled with [α ^32^P]dATP (3000 Ci/mmole; ICN products) and purified using a Qiagen Kit. Hybridizations were allowed to proceed in 0.5 M Na_2_HPO_4_-NaH_2_PO_4 _plus 7% SDS at 65°C for 16–18 h at 65°C. After probing, filters were washed once with 2 × SSC (1 × SSC = 0.15 M NaCl, 0.015 M sodium citrate)/0.1% SDS for 30 min at 65°C, and twice with 0.1 × SSC/0.1%SDS for 30 min at 65°C and then placed against X-ray film.

### Circular permutation assays

Double-stranded oligonucleotides containing the 5' and 3' ITR (Table 3, 5'ITRup+5'ITRdw and 3'ITRup+3'ITRdw) were cloned in the *Sma*I restriction site of pBS II SK+ (Stratagene), giving the p5' and p3' constructs. Six 113-bp fragments containing the 3'ITR or 5'ITR at various positions were amplified by PCR from p3' or p5' and six pairs of oligonucleotides (Table 3) as primers: O1+O1R; O2+O2R; O3+O3R; O4+O4R; O5+O5R; O6+O6R. Each fragment was purified from agarose gel, and ^32^P-end-labeled using a T4 polynucleotide kinase.

Binding reactions were carried out in 50 mM NaCl, 0.5 mM DTT, 10 mM Tris pH9, 5% glycerol, 5 mM MgCl_2 _and 100 ng of BSA. Each 20-μl reaction contained 0.2 pmol of labeled probes, and 400 nM purified Tpase [22]. The mixtures were incubated at 4°C, for 15 minutes, and then samples were loaded onto a 6% non-denaturing polyacrylamide (30:0.93) gel in 0.25 × TBE. The gel was run at 4°C and 200 V for 2 hours, dried and exposed to film overnight. At 4°C, two main complexes are expected, as described elsewhere [3,22]: SEC1 and SEC2, single-end complexes 1 and 2.

The relative mobility of each fragment (Rf) was defined as the migration distances of the bound complexes divided by the migration distance of free DNA. The Rf of each probe was plotted as a function of the position of the center in each probe to map the center of protein-induced bending. The apparent bend angle, α, was computed using the following equation [21]:

$$\frac{\mu_{\text{i}}}{\mu_{\text{j}}}=\frac{{\left[1-(2\chi_{\text{i}}/\text{L})(1-\cos\alpha )+2{\left(\chi_{\text{i}}/\text{L}\right)}^{2}(1-\cos\alpha )\right]}^{0.5}}{{\left[1-(2\chi_{\text{i}}/\text{L})(1-\cos\alpha )+2{\left(\chi_{\text{i}}/\text{L}\right)}^{2}(1-\cos\alpha )\right]}^{0.5}}$$

In which μ_i _and μ_j _are the Rf values of the complexes with proteins bound to a centrally positioned ITR and a peripherally positioned ITR respectively, χ_i_/L and χ_j_/L are the fractional distances (*i.e*., the distance between the position of the bending center and the left end of the fragment divided by the length of the probe) of the same probes, and α is the angle of the bend.

### Statistical analyses

All the data used for graphic representation corresponded to mean values obtained from 5–9 experiments. The differences between samples were analyzed using a non-parametric Wilcoxon/Kruskal-Wallis test with a significance threshold of at least 0.05 after checking the normality of each sample with a Shapiro-Wilk test to a significance threshold of 0.05.

## Abbreviations

ITR: inverted terminal repeat; SEC: single end complexes; PEC: paired ends complexes. EMSA: electrophoretic mobility shift assay. Tpase: transposase.

## Authors' contributions

The study was conceived and designed by CAG and YB. Transposition assays in bacteria (length dependence and GC content) were carried out by LS and GJ. BB carried out *in vitro *transposition and excision assays. FRB and YB studied minute *Mos1 *transposition. Circular permutation assays were carried out by CAG. The authors, in respect with their own contribution, analyzed the data. CAG and YB wrote the manuscript with input from FRB. All authors have read and approved the final manuscript.

## Supplementary Material

Additional File 1 **Sequences of DNA stuffer used in the study.** this file provides all the transgene sequences used in the study. Click here for file
