# Supplementary material for: Factors acting on Mos1 transposition efficiency
Source: BMC Mol Biol. 2008 Nov 26;9:106. doi: 10.1186/1471-2199-9-106 (PMC2642840; doi:10.1186/1471-2199-9-106)
Supplement: Additional File 1 — Sequences of DNA stuffer used in the study. this file provides all the transgene sequences used in the study. [file 1471-2199-9-106-S1.pdf]

Sequences of DNA stuffer used in the study

Minute Mos1 transposon sequence (3'ITR are in gray box)
TA-TCAGGTGTACAAGTATGAAATGTCGTTTGATATCAAGCTTGAATTCGCTGCAATAA
ACAAGTTAACAACAACAATTGGATCCACTAGTTCTAGAAAACGACATTTCATACTTGTAC
ACCTGA-TA

Tetracycline 1191 bp **(61.4% GC / 37 TA)**
ATGAAATCTAACAATGCGCTCATCGTCATCCTCGGCACCGTCACCCTGGATGCTGTAGGC
ATAGGCTTGGTTATGCCGGTACTGCCGGGCCTCTTGCGGGATATCGTCCATTCCGACAGC
ATCGCCAGTCACTATGGCGTGCTGCTAGCGCTATATGCGTTGATGCAATTTCTATGCGCA
CCCGTTCTCGGAGCACTGTCCGACCGCTTTGGCCGCCGCCCAGTCCTGCTCGCTTCGCTA
CTTGGAGCCACTATCGACTACGCGATCATGGCGACCACACCCGTCCTGTGGATCCTCTAC
GCCGGACGCATCGTGGCCGGCATCACCGGCGCCACAGGTGCGGTTGCTGGCGCCTATATC
GCCGACATCACCGATGGGGAAGATCGGGCTCGCCACTTCGGGCTCATGAGCGCTTGTTTC
GGCGTGGGTATGGTGGCAGGCCCCGTGGCCGGGGGACTGTTGGGCGCCATCTCCTTGCAT
GCACCATTCCTTGCGGCGGCGGTGCTCAACGGCCTCAACCTACTACTGGGCTGCTTCCTA
ATGCAGGAGTCGCATAAGGGAGAGCGTCGACCGATGCCCTTGAGAGCCTTCAACCCAGTC
AGCTCCTTCCGGTGGGCGCGGGGCATGACTATCGTCGCCGCACTTATGACTGTCTTCTTT
ATCATGCAACTCGTAGGACAGGTGCCGGCAGCGCTCTGGGTCATTTTCGGCGAGGACCGC
TTTCGCTGGAGCGCGACGATGATCGGCCTGTCGCTTGCGGTATTCGGAATCTTGCACGCC
CTCGCTCAAGCCTTCGTCACTGGTCCCGCCACCAAACGTTTCGGCGAGAAGCAGGCCATT
ATCGCCGGCATGGCGGCCGACGCGCTGGGCTACGTCTTGCTGGCGTTCGCGACGCGAGGC
TGGATGGCCTTCCCCATTATGATTCTTCTCGCTTCCGGCGGCATCGGGATGCCCGCGTTG
CAGGCCATGCTGTCCAGGCAGGTAGATGACGACCATCAGGGACAGCTTCAAGGATCGCTC
GCGGCTCTTACCAGCCTAACTTCGATCACTGGACCGCTGATCGTCACGGCGATTTATGCC
GCCTCGGCGAGCACATGGAACGGGTTGGCATGGATTGTAGGCGCCGCCCTATACCTTGTC
TGCCTCCCCGCGTTGCGTCGCGGTGCATGGAGCCGGGCCACCTCGACCTGA

Tetracycline [TA-rich] 1191 bp **(87 TA)**
ATGAAATCTAACAATGCGCTAATAGTCATCCTAGGCACCGTAACCCTAGATGCTGTAGGC
ATAGGCTTAGTTATGCCGGTACTGCCGGGCCTATTACGGGATATCGTCCATTCCGACAGC
ATCGCCAGTCACTATGGCGTGCTGCTAGCGCTATATGCGTTTATGCAATTTCTATGCGCA
CCCGTACTCGGAGCACTGTCCGACCGCTTTGGCCGCCGCCCAGTCCTGCTCGCTTCGCTA
CTAGGAGCCACTATCGACTACGCGATAATGGCGACCACACCCGTACTATGGATACTATAC
GCCGGACGCATAGTGGCCGGCATAACCGGCGCCACAGGTGCGGTAGCTGGCGCCTATATC
GCCGACATTACCGATGGGGAAGATCGGGCTCGCCACTTCGGGCTAATGAGCGCTTGTTTC
GGCGTGGGTATGGTGGCAGGCCCCGTAGCCGGGGGACTATTAGGCGCCATATCCTTACAT
GCACCATTCCTAGCGGCGGCGGTGCTAAACGGCCTCAACCTACTACTGGGCTGCTTCCTA
ATGCAGGAGTCGCATAAGGGAGAGCGTCGACCGATGCCCTTAAGAGCCTTCAACCCAGTA
AGCTCCTTCCGGTGGGCGCGGGGCATGACTATCGTAGCCGCACTTATGACTGTATTCTTT
ATAATGCAACTCGTAGGACAGGTACCGGCAGCGCTCTGGGTCATATTCGGCGAGGACCGC
TTTCGCTGGAGCGCTACGATGATAGGCCTGTCGCTAGCGGTATTCGGAATCTTACACGCC
CTCGCTCAAGCCTTCGTAACTGGTCCCGCCACCAAACGTTTCGGCGAGAAGCAGGCCATT
ATCGCCGGCATGGCGGCCGACGCGCTGGGCTACGTCTTGCTGGCGTTCGCGACGCGAGGC
TGGATGGCCTTCCCCATTATGATACTTCTCGCTTCCGGCGGCATCGGGATGCCCGCGTTA
CAGGCCATGCTATCCAGGCAGGTAGATGACGACCATCAGGGACAGCTACAAGGATCGCTC
GCGGCTCTTACCAGCCTAACTTCGATAACTGGACCGCTGATAGTCACGGCGATATATGCC
GCCTCGGCGAGCACATGGAACGGGTTGGCATGGATTGTAGGCGCCGCCCTATACCTAGTC
TGCCTACCCGCGTTACGTCGCGGTGCATGGAGCCGGGCCACCTCGACCTGA

Tetracycline [TA-poor] 1191 bp **(14 TA)**
ATGAAATCAAACAATGCGCTCATCGTCATCCTCGGCACCGTCACCCTGGATGCTGTTGGC
ATTGGCTTGGTCATGCCGGTCCTGCCGGGCCTCTTGCGGGACATCGTCCATTCCGACAGC

ATCGCCAGTCACTATGGCGTGCTGCTTGCGCTTTATGCGTTGATGCAATTTCTTTGCGCA
CCCGTTCTCGGAGCACTGTCCGACCGCTTTGGCCGCCGCCCAGTCCTGCTCGCTTCGCTT
CTTGGAGCCACAATCGACTACGCGATCATGGCGACCACACCCGTCCTGTGGATCCTCTAC
GCCGGACGCATCGTGGCCGGCATCACCGGCGCCACAGGTGCGGTTGCTGGCGCCTACATC
GCCGACATCACCGATGGGGAAGATCGGGCTCGCCACTTCGGGCTCATGAGCGCTTGTTTC
GGCGTGGGAATGGTGGCAGGCCCCGTGGCCGGGGGACTGTTGGGCGCCATCTCCTTGCAT
GCACCATTCCTTGCGGCGGCGGTGCTCAACGGCCTCAACCTTCTTCTGGGCTGCTTCCTT
ATGCAGGAGTCGCACAAGGGAGAGCGTCGACCGATGCCCTTGAGAGCCTTCAACCCAGTC
AGCTCCTTCCGGTGGGCGCGGGGCATGACAATCGTCGCCGCACTAATGACTGTCTTCTTT
ATCATGCAACTCGTTGGACAGGTGCCGGCAGCGCTCTGGGTCATTTTCGGCGAGGACCGC
TTTCGCTGGAGCGCGACGATGATCGGCCTGTCGCTTGCGGTTTTCGGAATCTTGCACGCC
CTCGCTCAAGCCTTCGTCACTGGTCCCGCCACCAAACGTTTCGGCGAGAAGCAGGCCATT
ATCGCCGGCATGGCGGCCGACGCGCTGGGCTACGTCTTGCTGGCGTTCGCGACGCGAGGC
TGGATGGCCTTCCCCATTATGATTCTTCTCGCTTCCGGCGGCATCGGGATGCCCGCGTTG
CAGGCCATGCTGTCCAGGCAGGTTGATGACGACCATCAGGGACAGCTTCAAGGATCGCTC
GCGGCTCTCACCAGCCTTACTTCGATCACTGGACCGCTGATCGTCACGGCGATTTATGCC
GCCTCGGCGAGCACATGGAACGGGTTGGCATGGATTGTTGGCGCCGCCCTTTACCTTGTC
TGCCTCCCCGCGTTGCGTCGCGGTGCATGGAGCCGGGCCACCTCGACCTGA

Kanamycin 1185 bp
ATGAGCCATATTCAACGGGAAACGTCTTGCTCTAGGCCGCGATTAAATTCCAACATGGAT
GCTGATTTATATGGGTATAAATGGGCTCGCGATAATGTCGGGCAATCAGGTGCGACAATC
TATCGATTGTATGGGAAGCCCGATGCGCCAGAGTTGTTTCTGAAACATGGCAAAGGTAGC
GTTGCCAATGATGTTACAGATGAGATGGTCAGACTAAACTGGCTGACGGAATTTATGCCT
CTTCCGACCATCAAGCATTTTATCCGTACTCCTGATGATGCATGGTTACTCACCACTGCG
ATCCCCGGGAAAACAGCATTCCAGGTATTAGAAGAATATCCTGATTCAGGTGAAAATATT
GTTGATGCGCTGGCAGTGTTCCTGCGCCGGTTGCATTCGATTCCTGTTTGTAATTGTCCT
TTTAACAGCGATCGCGTATTTCGTCTCGCTCAGGCGCAATCACGAATGAATAACGGTTTG
GTTGATGCGAGTGATTTTGATGACGAGCGTAATGGCTGGCCTGTTGAACAAGTCTGGAAA
GAAATGCATAAACTTTTGCCATTCTCACCGGATTCAGTCGTCACTCATGGTGATTTCTCA
CTTGATAACCTTATTTTTGACGAGGGGAAATTAATAGGTTGTATTGATGTTGGACGAGTC
GGAATCGCAGACCGATACCAGGATCTTGCCATCCTATGGAACTGCCTCGGTGAGTTTTCT
CCTTCATTACAGAAACGGCTTTTTCAAAAATATGGTATTGATAATCCTGATATGAATAAA
TTGCAGTTTCATTTGATGCTCGATGAGTTTTTCTAAGAATTAATTCATGAGCGGATACAT
ATTTGAATGTATTTAGAAAAATAAACAAATAGGGGTTCCGCGCACATTTCCCCGAAAAGT
GCCACCTAAATTGTAAGCGTTAATATTTTGTTAAAATTCGCGTTAAATTTTTGTTAAATC
AGCTCATTTTTTAACCAATAGGCCGAAATCGGCAAAATCCCTTATAAATCAAAAGAATAG
ACCGAGATAGGGTTGAGTGTTGTTCCAGTTTGGAACAAGAGTCCACTATTAAAGAACGTG
GACTCCAACGTCAAAGGGCGAAAAACCGTCTATCAGGGCGATGGCCACTACGTGAACCAT
CACCCTAATCAAGTTTTTTGGGGTCGAGGTGCCGTAAAGCACTAA

Bsr gene 423 bp
ATGAAGACCTTCAACATCTCCCAGCAGGATCTAGAATTAGTAGAAGTAGCGACAGAGAAG
ATTACAATGCTTTATGAGGATAATAAACATCATGTGGGAGCGGCAATTCGTACGAAAACA
GGAGAAATCATTTCGGCAGTACATATTGAAGCGTATATAGGACGAGTAACTGTTTGTGCA
GAAGCCATTGCGATTGGTAGTGCAGTTTCGAATGGACAAAAGGATTTTGACACGATTGTA
GCTGTTAGACACCCTTATTCTGACGAAGTAGATAGAAGTATTCGAGTGGTAAGTCCTTGT
GGTATGTGTAGGGAGTTGATTTCAGACTATGCACCAGATTGTTTTGTGTTAATAGAAATG
AATGGCAAGTTAGTCAAAACTACGATTGAAGAACTCATTCCACTCAAATATACCCGAAAT
TAA

Sh-ble gene 375 bp
ATGGCCAAGTTGACCAGTGCCGTTCCGGTGCTCACCGCGCGCGACGTCGCCGGAGCGGTC
GAGTTCTGGACCGACCGGCTCGGGTTCTCCCGGGACTTCGTGGAGGACGACTTCGCCGGT
GTGGTCCGGGACGACGTGACCCTGTTCATCAGCGCGGTCCAGGACCAGGTGGTGCCGGAC
AACACCCTGGCCTGGGTGTGGGTGCGCGGCCTGGACGAGCTGTACGCCGAGTGGTCGGAG
GTCGTGTCCACGAACTTCCGGGACGCCTCCGGGCCGGCCATGACCGAGATCGGCGAGCAG
CCGTGGGGGCGGGAGTTCGCCCTGCGCGACCCGGCCGGCAACTGCGTGCACTTCGTGGCC
GAGGAGCAGGACTGA

Sh-ble gene [no TA] 375 bp
ATGGCCAAGTTGACCAGTGCCGTTCCGGTGCTCACCGCGCGCGACGTCGCCGGAGCGGTC
GAGTTCTGGACCGACCGGCTCGGGTTCTCCCGGGACTTCGTGGAGGACGACTTCGCCGGT
GTGGTCCGGGACGACGTGACCCTGTTCATCAGCGCGGTCCAGGACCAGGTGGTGCCGGAC
AACACCCTGGCCTGGGTGTGGGTGCGCGGCCTGGACGAGCTGTTCGCCGAGTGGTCGGAG
GTCGTGTCCACGAACTTCCGGGACGCCTCCGGGCCGGCCATGACCGAGATCGGCGAGCAG
CCGTGGGGGCGGGAGTTCGCCCTGCGCGACCCGGCCGGCAACTGCGTGCACTTCGTGGCC
GAGGAGCAGGACTGA

Puro gene 662 bp
ATGACCGAGTACAAGCCCACGGTGCGCCTCGCCACCCGCGACGACGTCCCCCGGGCCGTA
CGCACCCTCGCCGCCGCGTTCGCCGACTACCCCGCCACGCGCCACACCGTCGACCCGGAC
CGCCACATCGAGCGGGTCACCGAGCTGCAAGAACTCTTCCTCACGCGCGTCGGGCTCGAC
ATCGGCAAGGTGTGGGTCGCGGACGACGGCGCCGCGGTGGCGGTCTGGACCACGCCGGAG
AGCGTCGAAGCGGGGGCGGTGTTCGCCGAGATCGGCCCGCGCATGGCCGAGTTGAGCGGT
TCCCGGCTGGCCGCGCAGCAACAGATGGAAGGCCTCCTGGCGCCGCACCGGCCCAAGGAG
CCCGCGTGGTTCCTGGCCACCGTCGGCGTCTCGCCCGACCACCAGGGCAAGGGTCTGGGC
AGCGCCGTCGTGCTCCCCGGAGTGGAGGCGGCCGAGCGCGCCGGGGTGCCCGCCTTCCTG
GAGACCTCCGCGCCCCGCAACCTCCCCTTCTACGAGCGGCTCGGCTTCACCGTCACCGCC
GACGTCGAGGTGCCCGAAGGACCGCGCACCTGGTGCATGACCCGCAAGCCCGGTGCCTGA
CGCCCGCCCCACGACCCGCAGCGCCCGACCGAAAGGAGCGCACGACCCCATGGCGCGCCT
GA

Fragment 1 1256 bp **(50.8% GC)**
AAAAGATAGACAGTGTGATTAAAAGACTGGACGACATTAAAATTTTGTATCTGACACCCG
AACGGGTGGTGTCGTCGAACCAACTGATGGACGCGCTAGCAACTCGGGTCTGTCGAATAG
TGATAGACGAGGCACACTGTGTCTGTGAGTGGGGGCACGATTTTAGATCCGACTACCTAA
AACTCTCCGTCCTTCGAACTATTTTCCCGAGCGTGCCGATACTGGCTTGCACCGCGACGG
CGACCCCGCGCGTGCGCAGGGATATCGTTCGCTTGCTCGGCCTTCGCGAAAACTTCGAGA
CGTTCGTCGGTTCGTTCGACAGACCCAATCTAAAAATCAGCGTTTACAAAAAGACTCCTC
GTCACACACTGACCGTGGTGGACCTCGTGAAGGCGAGAGCCGGCTCTTCGATCGTATATT
GTCTTTCGAGACGCGAAACCGAGGCGGTCGCTAACGCTTTGAAATGCGCCGGATTTAGCG
CGGAGGCGTATCACGCGGCAATGACTCCCAAGAACCGCACTTCCGTGCAGAACAGATGGA
TGGCGAACGAGACCCGAGTGATATGTTCGACGATAGCGTTCGGGATGGGCATCGATAAAC
CCGACGTGCGGTTGGTGATACATCACAGTATGCCCAAATCACTGGACGGTTATTATCAAG
AGATAGGAAGGGCCGGTCGCGACGGAAAACTGTCGGAGTGTATCGCCTTTTACGGCGCCA
ACGACGTCTCGAGATACAAATATCTTTTCAGGATCAGCCAGAGCGACAATCCCAGGAACA
ATAAGATGCTGTCCACAATGTACACGTTCTGTACCAACACCTCCATTTGTCGCAGACGTC
AAATATTGCGGTACTTCGACGAGACGGTGGGCGACAGGGTCTTATGCGATTCTGTAAATT
CTTGCGACAACTGTAAATTTTATTTTGGTTTCTTACGCGCGCGCGTTAGAAATCACATGT
ATGAATATTTCTGGCTGTCCGCCCATACGCTCTTGTCGTTGACATTGACGACTATCATGT
CGGGGAGTTCTCTGACGATAAAAACACCATAAGCGGTTTCTACGATACTGTACGGCACTT
CTACGACTTCGCCCGACGACGTCGCCACCAGAACCTTGTCGCTGTCGTAGAACATGAGCG
AATTGGCCCCTAGATTTATTCTTTTACCGAGAAGATCAAATTCGGCCACGAGCGGCCTTC
TGAAATCGTTTCTCATCATGTATTGGACGTCCGTCACGACGAGATCCGGGGAATTC

Fragment 6 1100 bp **(43.3% GC)**
TGTCACCGAATCGTATATTGGTTATAGACATGGCGCCTTCCGTGATAACTGACACACACA
CACACACACACACAATCTATTGTCAACCATCAATTCCTTTTGATCGACACATCGGGGTCA
GTTGGTTTTTTGCTTTTTTAGTGTGATAATTTCGATTTTTTACTACGATACAGTGGGTCG
TGAAAAGGGACTTTTGATTATTAATCAACGAGCACTCGCACTACCCCATCGAACGATTTC
ATCGGACGGAACGTGTTGTGACGGCGGTGAATATCTACTTGTCGACACGTTTACAAAGTT
AAACTTTAAAGTGCAAACGATACGTAACGCATCGAAGATCCTGCTCCAAACGACCCTGCG
CAACTACATCGAAAAGAACGTGAAACAGGTCGCCTGCTACTTTGTCGTGGTGCTCAACGA
CGGTAACGACGGCCACAACATACTCACAACCCCCGGAACTACTCATTGTCCCAGCTGTAT
GCATTGTTCACGTTGTACAGGGTGCGCGCGATACCGAAAGTGTTTCTCATACAATCGTGT
TTGGGTGCTAAGATAGATCCCAGTCATTGCGATCGAGCGTCGTGTCAATGTGACCAAGAA
GAAGACGCGCATCCCACGTCCGTGTGTCACGACATCTTCGTCAACACAGTTCGTCGTGTG
ATACACGCATGCAGTCGAAAATCTAAGGGGTCGACAACAACTACCGAAACTAAATGTTGG
GATGTGGCGAGAGGGGTTCTCACATGACGGCACAGGGAAGAAATTATCATAGTTTATTTG
CGTATGGAAGGGTATGTGAGGTAGGGTGACACAAAGTGTGGGTGTTTGATGATTGAGAAA
TTTTGAAAAAATCTCATAAAATAGGGTACTAGAAGGAGTGTACACACAAGGATAAGGATG
GTACAAAAGGAAATGCAAATCAGGGATCCAAAATATGTACCAATTGTGGAAATGAATTGT
ACAAAACTGTTATTTCTGGGGGAGGAGAACAATATAATAATGGAAGAGTATTAGATTTGG
GTTGGGGTGGGGTGGTCTGTTTGTGAAATAAAAGTCTGGGCTTTTTACTGCTAAAATTTA
TTGAAATGGATTCGAAAGAAAGGGAATTCA

Fragment 46 1056 bp **(50% GC)**
AAGGACAATCCCGGCTGTGCACAAGTCGACAACATGTACTACATACAACAGCTGGTCAAC
CCTCTCACTAAGGTTTCCGAAGCGGTGTGGCGCCGCAGTAACAGCGTTAACATTGCCATC
GCGCCGTTCATCTATTACAATAAGGTTTTAGCTCAATTGTCAACTAGGTTTAAGATTTCG
CAATAAAACTACAATGGGTACAAAACAAAATTTGTTTTATTTCATTTTCTCAAATACACG
ATTCGATCCACCAGGGTTTCCTCGGTATATTCCTAGAAGTCCACATGCGATCCGCACATG
ATGTAGGTCGCATCTCGTCGAACCGTTGCGCCGACAATCTATTCATCTGTACGTGCATCA
ATGTGATGAAGTCATCGAGACACTGTGCAAAATCAACGGGGCAGCTGCCGACGATGCGGT
CCGTCAACGTGCCGACAATCGTGCACTTGTGACGCTCAAGTCATCGACGTAGTGTCTAAC
GGTAGCGGCCAGCAGTTCACGATCTCGTCCGAGTGTGGGCGTGTTGATACCGTGGTCGGT
CTCAGCGCGTACACGAGCGGTGTGATACGACGATTGTCGTTCCTGATCGTGCCGTTCAGG
ATTCTCGAGTGAGTCTTGTGTATTTCATGGTAGTTACGTAGATATGCACCCTCGGTGGGA
TCGATGACGCTAGTCAAACGCATGGTGGATGGGTAGAGACACGGCCACCGAATGGTGATC
ACGCACACCGCCTATGGTTGCATTATTCCGTAAGTGCCGGGTACTCGTGCCATATCAACT
CGGCACACACCCACACCGACAGATGTTTCGAAGTCGCTACTGCGCAAGCCTTCTAGCGCA
GCGTGTAAAATAGTTATGGAATTGGAATGTAATGCGTTCACGGGCCGCTGTTGCTCGATC
AAGTCGATCAGCTCGTCCACCAGTTGCAGTCGATATCTTCTCGGGTCCGATGTCTGTAGC
CAAGTCGATGAAATCGTGCGTGGTACGTGCGGAAATGCTTCCCCACTCCAATCTATGGTG
CCGTATTCGAAAACACACCTACACTTGCCGATACAC

Fragment 108 1123 bp **(37.7% GC)**
TGGAGCGTTACCGTTTATGCCAATCGCAATGTGCACAACATACATGGGAAGAAAAAGTAT
TCTTGAAGTTGTAGAACACGTGAAAGATTATGGAGGAACAGTGGTGTACGGGGACACGGA
CTGCCTTTGTCGAACCACTCCTGTTTTAATTCGAAAAGACGACAGCGATGTTTTTTTTAC
AACTGTGGATGAGTTAAGTAAAAATGACTGGGAGTACATAAATCCCAACAAAGACATCTC
GTCCCCCAAAGAAAATTACGAAATATGGAGTGACACAGGATTTACAAAGATCGAAAATGT
TGTAAGATGTAAAATAGACGAGCCCCTAAAACGTGTAACTACACATGTGGGCTCTGTAAT
TTGTTCCGACAACCACTCCTTACTCACCGAAAATTTGACAGGTGTAACACCAAATGATGT
TGTTATAGGGGATAGATTGTGTGTAAGAGAACTTCCGTTACAACAAGATACACCACAAGT
TCCAATATATATAAATAGACTTACCGCAGAGAGGATCCAAATGTATGAAATCCCAGAAGT
TTCTTACCTAAACGTGTCTGCCAAAATGGCTTTTGTATGGGGATTATTTTTTGCGGACGG
TGGTGCCGGTATTTACAAACAAAGCAAATCTAATTGTTTGTCTAATTTCACATGGGCTAT

CAATAAAGCTCTGTCACGTTATGAAATGGGAGGATCATGTGTCATGTGAACATGATCCAA
AAATCATCAGAAAAAAAGAATTAACAGTCAAAATAGACGAATTGAAAAGAAAGATTAAGG
ATGAAAAGGACAGGGGTAGGAAGGATAAGTTAAAAATAGACTTGGCGTCCTTAACAAAAG
AAAGGTCTAACATAACAAAAAAACTTAGTAAAAGTGTTATGTGTGCTAAAAGAAGTTATA
GATTTTTAAAAGAGCCTCGAGGAGTTCTTCCGACAATCATAAAGGATCTTCTGGACGCCA
GAAAAAACACTCGTGCGGAAATAAAAATATGGCAAAATAAACTTAAATCAGCAGGTGGTC
TTGAAGAACAAGAAATCGCAAAATCAATGATCCAAATTCTGGACCAAAGACAAAATTCGT
ACAAAGTCAGTGCTAACTCAATGTATGGAGCAACCGGTGTTAG

Fragment 116 1141 bp **(44.3% GC)**
GCGGAGATTTTATAAGCGAGCTGTGACTTATCGAGTATATCTCGAGCWGAGGATCGTAAC
TTCTTCATTTCAGCTCGTACCTTCCTGCGAGCGTCTAAGAAGTATTCAACCAATCTGGTA
GTGTCTCATGCGAGTTTTCAGTATACGAACTCGTTGTTTTGCGCACAGTCTAGAACCACC
AGACATTCTTGACTTTAGCACTTTCAATCGGATGGCGGCCCGCTGTAAATCAACCGACGA
CAGCTTGATTGATGGTATCGGAGAACCGTTGTAATTATCATCGGTAAAACCATTACATTC
CGAGTAATCTTCAACGTCGTCAACTGGCTTACGCGTTGTACTTGTCGGCTTGAAGTACTT
TGTAATCGGCTGAACCCCGATGCATTTTCGCTTCTCGACTTTTGCTAGATCTTCGAATAT
TGACTTTAGTTTTTCGCGCTCTTTTATGTTTGGATCGTGCTCGCAACCAACGTGATCCTC
CCATTCTACAATTTCAAAGTTTTCGTCGGTGTAGTCACTGTGTGCATCGTCGTCGATGAC
CGTACTGTAGCAGAGATTATAAGCCATCATCAACGTCGGATATATCCGACGTTGATGATG
GTTATAATCTCTGCTACAGTACGGTCATCGACGACGATGCACACAGTGACTACACCGACG
AAAACTTTGAAATTGTAGAATGGGAGGATCACGTTGGTTGCGAGCACGATCCAAACATAA
AAGAGCGCGAAAAACTAAAGTCAATATTCGAAGATCTAGCAAAAGTCGAGAAGCGAAAAT
GCATCGGGGTTCAGCCGATTACAAAGTACTTCAAGCCGACAAGTACAACGCGTAAGCAGT
TGACGACGTTGAAGATTACTCGGAATGTAATGTTTTACCGATGATAATTACAACGGTTCT
CCGATACCATCAATCAAGCTGTCGTCGGTTGATTTACAGCGGGCCGCCATCCGATTGAAA
GTGCTAAAGTCAAGAATGTCTGGTGGTTCTAGACTGTGCGCAAAACAACGAGTTCGTATA
CTGAAAACTCGCAGAGGACTACTACCAGATTTGGTTGAATACTTCTTAGACGCTCGCAGG
AAGGTACGAGCTGAAATGAAGAACGTTAGCGATCCTCTAGCTCGAGATATACTCGATAAG
T

Fragment 193 1127 bp **(56.5% GC)**
ACATCGAACACGCCAAGGCGATCGCCGATGAACAAGCGGCTCGTGCCCTTGTACGTTGCG
ATGTACAGTAACTGGTCGAATACAAACGCACCGACTCGAGCCACCATGGACATCGAAGCG
ATACGCGGACGTTACACCGTCTCCGTACCGGAGAGCATGGAACGTCCGATGGTGATTCTG
TTCAACGACTTGACAATCTCCGAGGATTTGGTGGTGTTCGCGACGCACGGTGAGTTGATC
AAGTACGACACTCGATACCCGCCGTCGTCGGACGCAGCGCTGGCCGCCATGAGCAGTCGT
CGCGCCAGGAAGGGTGGCGAACGAGACAAACGCATCTCGTTGTACATGGTGCGACGCGGT
ATCTACTCGCCGGGCATGAACTACGACATAGCGCACGTACACCAGACTCCCGGCGTGGGC
TACGTGATCACGGTGATCAGTAATAACGCGCCCACCGTCGACGTGCCTCGAAGTCTAGTC
ACCGCACTCGGCATGGGCGGTGACGACGATCAGGTGGTGGTGAGCGATGTCGGCGGCGGT
GTTACATACACCGCGTCGTTCGTCCTAAACGACATCACGTTCGTCACAGTACCTGCAACA
TTACACGTTGCTCAAGTGTACCGACAAGGCGACGTGTATATTCGTCGACGAAACGCATCT
GATGAAGTACGCGCGCCCTTGGCGTCGATACACAATCGCCAGTCCGCGATTCATATACGA
GTACAATAATTTCACGGTGAAGTTTAGCGCGACCGTGGAGAGCACGCGCAGGGACGTGGC
TCGAGTGTCGCCCTATTGCCGTATCCGTATCTACGATGCGCCCAACACGCAGATCATGTA
CGGCATGGCGAACGACATAACCGCACACATGATCGAGTACAAGAGTAGGGAGCGTTCGAT
CCCGTCTCTGTACGCCGAGAATCTACACAACGATCAGCTGTTGCGGATCAACGTGCGCAT
AATGCGTGACTCGAAACCGTTAAACGCGAACCCGGCTACCTGGCTCGGCAACACCGACAG
GTCCGTAGAAAACTGCAAGCGCAAAAGACCGGCACCAACGCAGACGTACGCTCCCTGATC
GGTGTCAACAACTACGCCAGACAATGTGCACGCCTACCTACAGCCGT

Fragment 240 1134 bp **(46.8% GC)**
TCGCCGTTGCTGTCGCCTACTGTCGGCGCCTTGTTGATGCTGTTGTTTCTCGATCGAGTG
GTTGGTGAGTGCGTTATCGGCAATTTCGCTGAAACTTTTCAAATCTTCCACACCCTCCGT
CAACGTTCTCCACGTACCGCGTTGGCCGTTGAATCGTCGGAACAGAGCCAGGGCGGCGAA
CAATGCCGCGATCGCTATGATCGTGTACTTGTTGCTGAACACCGATTCGGCGTCGTTCAT
AGCCCTCTTCCAAGCCAACCAAGCACCCATCAATGCTACGGACACGATAAGCACATGCTT
AATTTCCATTTACCCAGTCGAAACAGCGAAGGACGGTAACGGATACAAAGAAAATAAAAG
ACACATTTTTAGATTAACCATTTATTGACACACAGTGTTACCACAAAGATCGCAATAACC
GTCGACGTCACCATCACGTTTGGGACAGTACTGTAGGAATCGCACATCGACCGCTATACT
GTTCGTACAGTACAGGAAGGCAAACGTAAAGTCCACAAACGACCTCGGAGAGTTGACTGC
AAAAAATAACACCATCTGGTTCCGTAAATGCCAGAGAGATTCACTCGCCGCTCCGGTTAT
GTCGCGCAATTGCACCAGGTTGGATCGTGGAAATTGTAAGTATGTTTTGTTGAAACGTTT
CATTATCACCGTCGTATAGTTACCACGGTGCACTCGGATCTTTGCACTGACCACTCGACG
AATCACACCGGTGCGTGATCTTCGCAACAGACAATATTCATCACCCGTACTGGGATCTAT
GAAGACGTCGAATATTGTCGAGTAAAAGTTATGCGTGTCGTATGCACAACCCAGCGAAGG
TCTGGAGATAATCGAACGCATCATCATCTTCTCACCACCACAATGACCGATGAACGAATA
ATACCAATCGTCGTACTGCATCTCGCGATTAACTGTCCTCGCCCGTTTAATACATTTCGA
TTTATATGTTTTTATGCTGTAACACTAATGTGTATGTTACACGCGAACAGAAACGTGACT
TCTTGAACTGCGACCGGTCGGTCGAGCGAGCGATCAATCGTAACGTTGACTGTATTGTAT
TATTGTCGAAACTACACTGTCCTCGAATGTTTTGTATCTGTCCACCGTCGACGA

Fragment 289 1157 bp **(45% GC)**
TACGATCCCGGCGTAGTCTCCGATAACTCCGTACACGAATTGTCGCACAATCTTGGTACA
ATGCGGCGAACCCATGAGCGAACACCATAAAGCGCCCACGAAAGATTCCATCACGTCCTC
GAGTAACGATATCACGTCTCGATGTATGTGGGTGGAGGTCGTGTCTGTGTACACGGAATC
GGCACACACTTTCGGTTGACATTGAACGCCGGACGCGTTGTTGCGTTTGAATCTGTGTCC
GTACACTTGTAATTTTCTACCATCGACACCGGCGTACTGGCACCTTATGAACTTGTTCAA
TCCGATCGATACACAACACTTTGACAGATTCCACTTTCCCACGTAGATGCTGCGTAGTTT
CGTCATCACACCGATGTCTTGCTGAATGTTATGTATATCCGTGTAGTTTTCACGAATGTA
GTCTATTATGAAGCGGTTGATGACGGAATCACCGATGTACTCGAAGTATTCGTAGTTGAA
TATGTTGTCGTATTCTTTGGAGGTGAGCGCCGCTTGAAATTTATCCATATTACCCTCATC
ACGAAACGTGTCCAATAAATGTTTATAGTATTGCGCGTCGACGTTACCATATTCGATGAC
AATTTCGGCAATGTGTTCGGCGGTTATGATGACGGGGGTGGCACATCCGTTACCGTCTCC
TTGTGGTGACATATCGATTAAGTTGCCTTCATCCAGCGCCCAATGTAAAACCCTCGCTCT
CCAATGTCGACCGCTGTACTCGGCCGTGTAACGGTTGTGTGTAATGCAACGTTTCGGAAC
TAAGACAAATTTAGCGGTGGTGGTGGTGGTGGTTGTTCTTTTGAACGATGACCTTCTCTT
TTTGAAATGGTCGATTTTATTGAACGGTTTTTCCTTGACGTTCCGGTTGGCAGTTCTATA
ACGCAAGAGAAACGACTTATCAACGTGGTTATACTTTGACGTCCGTGAAACGACTGTGGT
GTTAGCATCAATTGTTGTTGTTGTACGGTTGTTGTCGTACGCTTGTTGTTGTGTTGTAAC
ATCGGTCTCATTGTTACAATTCATACTGAACGATATGTGATTTAATTAATACACACAAAC
ACGTGTCATGTGTACACGGGATGTTCCCATATACTGATGATGATATGTGGAAGGGGGAAT
TGAAACAACTCATTCGT

Fragment 523 1105 bp **(47.5% GC)**
CGACGACTCCCGGTAATGTGTCACCACCGTTGTTAACGACACCGACCTCGTTGGGTAGGC
GCGGATAACTGATCGCTGTGCTTCCACTCATTTACCATTGTGAGATGAAAATGGTTCAAG
CACAGTGTTCAGCTACAAAGCTATCAATGCTTATCGTATGCATCATCATCATGATTACAA
CCACGCTACCGGATCACGGTTGTATGGGTTTCGACGTTAAAAGCACAATAGTAGCCGCTT
TAGATCCGGTGACTTCGTTGATTAACCGCGTCAAATCGTCAATCGAAGACGTCAAGACCG
AGGTTAACACCGTTATAGAGGACGTTAAATCGTTGAAGAACGACGCCATCAACAAAGTCA
GCGAAGTGGTATCAACGACTAAATCGATGGCGGTGGAAATTAAACAATTCACTAATTTAA
CAACATTTATTCATAAACTTAGGAATGTAATAACAAAACAAGACATCTTAGACCTAGAGA
CGGGCGTTATAAATTTCATACTAGACTTGTCCGTACACCTGGCGGTTACCGTTCATAAAC
GTTGGCCGATGTTAGTGTACGCGGCGGCCGCATGTACCGCCCTTGTAGCTACCATAATCA

TCATCTTGACTATATCGATATGCGTGCTGTCGTGTCGACTACGAAGATTCGACGATGCCA
TCAAGGACACGGTTGAATGATGCGAGTACGTCTTCGCGTGGCAAGTTTCCGTTCAACACG
AAAACTGAACTATGGTTGTCGACGTATCGTTTCGTGTAGCATTCGACTCTTTCGAAATAC
TCGACGTCCAATGATTTCTCCGCCTCGCGTCCTCGTTCCTGAGCTCGGCGGTGACAGGTG
TCCGGTTTCGTGTCAATGTACACGACGTGGTCGATTCTCGGAATGTCATTCTCCAAACTG
CGAAACACATCCGTGTACACGTCGAATTGTTTGTCGGTGATCGAGCCTCGGTCTCGCATC
ATTCTGGTGAAGACGTCGTACGCCGACCACGGCGAGCGTTCCATGATAACGACTCGAGCT
CCGGTTCGCAGACATGTCTCGTACGAGTCCAACCACTTTTGATATTTACTCGTCAGCACT
TGCAGCTCGAAGGGGAAAGCGTATC

Tet-1 (56.3% GC)

ATGAAATCTAACAATGCGCTCATCGTCATCCTCGGCACCGTCACCCTGGATGCTGTAGGC
ATAGGCTTGGTTATGCCGGTACTGCCGGGCCTCTTGCGGGATATCGTCCATTCCGACAGC
ATCGCCAGTCACTATGGCGTGCTGCTAGCGCTATATGCGTTGATGCAATTTCTATGCGCA
CCCGTTCTCGGAGCACTGTCCGACCGCTTTGGCCGCCGCCCAGTCCTGCTCGCTTCGCTA
CTTGGAGCCACTATCGACTACGCGATCATGGCGACCACACCCGTCCTGTGGATCCTCTAC
GCCGGACGCATCGTGGCCGGCATCACCGGCGCCACAGGTGCGGTTGCTGGCGCCTATATC
GCCGACATCACCGATGGGGAAGATCGGGCTCGCCACTTCGGGCTCATGAGCGCTTGTTTC
GGCGTGGGTATGGTGGCAGGCCCCGTGGCCGGGGGACTGTTGGGCGCCATCTCCTTGCAT
GCACCATTCCTTGCGGCGGCGGTGCTCAACGGCCTCAACCTACTACTGGGCTGCTTCCTA
ATGCAGGAGTCGCATAAGGGAGAGCGTCGACCGATGCCCTTGAGAGCCTTCAACCCAGTC
AGCTCCTTCCGGTGGGCGCGGGGCATGACTATCGTCGCCGCACTTATGACTGTCTTCTTT
ATCATGCAACTCGTAGGACAGGTGCCGGCAGCGCTCTGGGTCATTTTCGGCGAGGACCGC
TTTCGCTGGAGCGCGACGATGATCGGCCTGTCGCTTGCGGTATTCGGAATCTTGCACGCC
CTCGCTCAAGCCTTCGTCACTGGTCCCGCCACCAAACGTTTCGGCGAGAAGCAGGCCATT
ATCGCCGGCATGGCGGCCGACGCGCTGGGCTACGTCTTGCTGGCGTTCGCGACGCGAGGC
TGGATGGCCTTCCCCATTATGATTCTTCTCGCTTCCGGCGGCATCGGGATGCCCGCGTTG
CAGGCCATGCTGTCCAGGCAGGTAGATGACGACCATCAGGGACAGCTTCAAGGATCGCTC
GCGGCTCTTACCAGCCTAACTTCGATCACTGGACCGCTGATCGTCACGGCGATTTATGCC
GCCTCGGCGAGCACATGGAACGGGTTGGCATGGATTGTAGGCGCCGCCCTATACCTTGTC
TGCCTCCCCGCGTTGCGTCGCGGTGCATGGAGCCGGGCCACCTCGACCTGAATGGAAGCC
GGCGGCACCTCGCTAA
AAAAGATAGACAGTGTGATTAAAAGACTGGACGACATTAAAATTTTGTATCTGACACCCG
AACGGGTGGTGTCGTCGAACCAACTGATGGACGCGCTAGCAACTCGGGTCTGTCGAATAG
TGATAGACGAGGCACACTGTGTCTGTGAGTGGGGGCACGATTTTAGATCCGACTACCTAA
AACTCTCCGTCCTTCGAACTATTTTCCCGAGCGTGCCGATACTGGCTTGCACCGCGACGG
CGACCCCGCGCGTGCGCAGGGATATCGTTCGCTTGCTCGGCCTTCGCGAAAACTTCGAGA
CGTTCGTCGGTTCGTTCGACAGACCCAATCTAAAAATCAGCGTTTACAAAAAGACTCCTC
GTCACACACTGACCGTGGTGGACCTCGTGAAGGCGAGAGCCGGCTCTTCGATCGTATATT
GTCTTTCGAGACGCGAAACCGAGGCGGTCGCTAACGCTTTGAAATGCGCCGGATTTAGCG
CGGAGGCGTATCACGCGGCAATGACTCCCAAGAACCGCACTTCCGTGCAGAACAGATGGA
TGGCGAACGAGACCCGAGTGATATGTTCGACGATAGCGTTCGGGATGGGCATCGATAAAC
CCGACGTGCGGTTGGTGATACATCACAGTATGCCCAAATCACTGGACGGTTATTTATCAAG
AGATAGGAAGGGCCGGTCGCGACGGAAAACTGTCGGAGTGTATCGCCTTTTACGGCGCCA
ACGACGTCTCGAGATACAAATATCTTTTCAGGATCAGCCAGAGCGACAATCCCAGGAACA
ATAAGATGCTGTCCACAATGTACACGTTCTGTACCAACACCTCCATTTGTCGCAGACGTC
AAATATTGCGGTACTTCGACGAGACGGTGGGCGACAGGGTCTTATGCGATTCTGTAAATT
CTTGCGACAACTGTAAATTTTATTTTGGTTTCTTACGCGCGCGCGTTAGAAATCACATGT
ATGAATATTTCTGGCTGTCCGCCCATACGCTCTTGTCGTTGACATTGACGACTATCATGT
CGGGGAGTTCTCTGACGATAAAAACACCATAAGCGGTTTCTACGATACTGTACGGCACTT
CTACGACTTCGCCCGACGACGTCGCCACCAGAACCTTGTCGCTGTCGTAGAACATGAGCG
AATTGGCCCCTAGATTTATTCTTTTACCGAGAAGATCAAATTCGGCCACGAGCGGCCTTC
TGAAATCGTTTCTCATCATGTATTGGACGTCCGTCACGACGAGATCCGGGGAATTC

Tet-6 (52.9% GC)

ATGAAATCTAACAATGCGCTCATCGTCATCCTCGGCACCGTCACCCTGGATGCTGTAGGC
ATAGGCTTGGTTATGCCGGTACTGCCGGGCCTCTTGCGGGATATCGTCCATTCCGACAGC
ATCGCCAGTCACTATGGCGTGCTGCTAGCGCTATATGCGTTGATGCAATTTCTATGCGCA
CCCGTTCTCGGAGCACTGTCCGACCGCTTTGGCCGCCGCCCAGTCCTGCTCGCTTCGCTA
CTTGGAGCCACTATCGACTACGCGATCATGGCGACCACACCCGTCCTGTGGATCCTCTAC
GCCGGACGCATCGTGGCCGGCATCACCGGCGCCACAGGTGCGGTTGCTGGCGCCTATATC
GCCGACATCACCGATGGGGAAGATCGGGCTCGCCACTTCGGGCTCATGAGCGCTTGTTTC
GGCGTGGGTATGGTGGCAGGCCCCGTGGCCGGGGGACTGTTGGGCGCCATCTCCTTGCAT
GCACCATTCCTTGCGGCGGCGGTGCTCAACGGCCTCAACCTACTACTGGGCTGCTTCCTA
ATGCAGGAGTCGCATAAGGGAGAGCGTCGACCGATGCCCTTGAGAGCCTTCAACCCAGTC
AGCTCCTTCCGGTGGGCGCGGGGCATGACTATCGTCGCCGCACTTATGACTGTCTTCTTT
ATCATGCAACTCGTAGGACAGGTGCCGGCAGCGCTCTGGGTCATTTTCGGCGAGGACCGC
TTTCGCTGGAGCGCGACGATGATCGGCCTGTCGCTTGCGGTATTCGGAATCTTGCACGCC
CTCGCTCAAGCCTTCGTCACTGGTCCCGCCACCAAACGTTTCGGCGAGAAGCAGGCCATT
ATCGCCGGCATGGCGGCCGACGCGCTGGGCTACGTCTTGCTGGCGTTCGCGACGCGAGGC
TGGATGGCCTTCCCCATTATGATTCTTCTCGCTTCCGGCGGCATCGGGATGCCCGCGTTG
CAGGCCATGCTGTCCAGGCAGGTAGATGACGACCATCAGGGACAGCTTCAAGGATCGCTC
GCGGCTCTTACCAGCCTAACTTCGATCACTGGACCGCTGATCGTCACGGCGATTTATGCC
GCCTCGGCGAGCACATGGAACGGGTTGGCATGGATTGTAGGCGCCGCCCTATACCTTGTC
TGCCTCCCCGCGTTGCGTCGCGGTGCATGGAGCCGGGCCACCTCGACCTGAATGGAAGCC
GGCGGCACCTCGCTAA
TGTCACCGAATCGTATATTGGTTATAGACATGGCGCCTTCCGTGATAACTGACACACACA
CACACACACACACAATCTATTGTCAACCATCAATTCCTTTTGATCGACACATCGGGGTCA
GTTGGTTTTTTGCTTTTTTAGTGTGATAATTTCGATTTTTTACTACGATACAGTGGGTCG
TGAAAAGGGACTTTTGATTATTAATCAACGAGCACTCGCACTACCCCATCGAACGATTTC
ATCGGACGGAACGTGTTGTGACGGCGGTGAATATCTACTTGTCGACACGTTTACAAAGTT
AAACTTTAAAGTGCAAACGATACGTAACGCATCGAAGATCCTGCTCCAAACGACCCTGCG
CAACTACATCGAAAAGAACGTGAAACAGGTCGCCTGCTACTTTGTCGTGGTGCTCAACGA
CGGTAACGACGGCCACAACATACTCACAACCCCCGGAACTACTCATTGTCCCAGCTGTAT
GCATTGTTCACGTTGTACAGGGTGCGCGCGATACCGAAAGTGTTTCTCATACAATCGTGT
TTGGGTGCTAAGATAGATCCCAGTCATTGCGATCGAGCGTCGTGTCAATGTGACCAAGAA
GAAGACGCGCATCCCACGTCCGTGTGTCACGACATCTTCGTCAACACAGTTCGTCGTGTG
ATACACGCATGCAGTCGAAAATCTAAGGGGTCGACAACAACTACCGAAACTAAATGTTGG
GATGTGGCGAGAGGGGTTCTCACATGACGGCACAGGGAAGAAATTATCATAGTTTATTTG
CGTATGGAAGGGTATGTGAGGTAGGGTGACACAAAGTGTGGGTGTTTGATGATTGAGAAA
TTTTGAAAAAATCTCATAAAATAGGGTACTAGAAGGAGTGTACACACAAGGATAAGGATG
GTACAAAAGGAAATGCAAATCAGGGATCCAAAATATGTACCAATTGTGGAAATGAATTGT
ACAAAACTGTTATTTCTGGGGGAGGAGAACAATATAATAATGGAAGAGTATTAGATTTGG
GTTGGGGTGGGGTGGTCTGTTTGTGAAATAAAAGTCTGGGCTTTTTACTGCTAAAATTTA
TTGAAATGGATTCGAAAGAAAGGGAATTCA

Tet-46 (56.2% GC)

ATGAAATCTAACAATGCGCTCATCGTCATCCTCGGCACCGTCACCCTGGATGCTGTAGGC
ATAGGCTTGGTTATGCCGGTACTGCCGGGCCTCTTGCGGGATATCGTCCATTCCGACAGC
ATCGCCAGTCACTATGGCGTGCTGCTAGCGCTATATGCGTTGATGCAATTTCTATGCGCA
CCCGTTCTCGGAGCACTGTCCGACCGCTTTGGCCGCCGCCCAGTCCTGCTCGCTTCGCTA
CTTGGAGCCACTATCGACTACGCGATCATGGCGACCACACCCGTCCTGTGGATCCTCTAC
GCCGGACGCATCGTGGCCGGCATCACCGGCGCCACAGGTGCGGTTGCTGGCGCCTATATC
GCCGACATCACCGATGGGGAAGATCGGGCTCGCCACTTCGGGCTCATGAGCGCTTGTTTC
GGCGTGGGTATGGTGGCAGGCCCCGTGGCCGGGGGACTGTTGGGCGCCATCTCCTTGCAT
GCACCATTCCTTGCGGCGGCGGTGCTCAACGGCCTCAACCTACTACTGGGCTGCTTCCTA
ATGCAGGAGTCGCATAAGGGAGAGCGTCGACCGATGCCCTTGAGAGCCTTCAACCCAGTC
AGCTCCTTCCGGTGGGCGCGGGGCATGACTATCGTCGCCGCACTTATGACTGTCTTCTTT

ATCATGCAACTCGTAGGACAGGTGCCGGCAGCGCTCTGGGTCATTTTCGGCGAGGACCGC
TTTCGCTGGAGCGCGACGATGATCGGCCTGTCGCTTGCGGTATTCGGAATCTTGCACGCC
CTCGCTCAAGCCTTCGTCACTGGTCCCGCCACCAAACGTTTCGGCGAGAAGCAGGCCATT
ATCGCCGGCATGGCGGCCGACGCGCTGGGCTACGTCTTGCTGGCGTTCGCGACGCGAGGC
TGGATGGCCTTCCCCATTATGATTCTTCTCGCTTCCGGCGGCATCGGGATGCCCGCGTTG
CAGGCCATGCTGTCCAGGCAGGTAGATGACGACCATCAGGGACAGCTTCAAGGATCGCTC
GCGGCTCTTACCAGCCTAACTTCGATCACTGGACCGCTGATCGTCACGGCGATTTATGCC
GCCTCGGCGAGCACATGGAACGGGTTGGCATGGATTGTAGGCGCCGCCCTATACCTTGTC
TGCCTCCCCGCGTTGCGTCGCGGTGCATGGAGCCGGGCCACCTCGACCTGAATGGAAGCC
GGCGGCACCTCGCTAA
AAGGACAATCCCGGCTGTGCACAAGTCGACAACATGTACTACATACAACAGCTGGTCAAC
CCTCTCACTAAGGTTTCCGAAGCGGTGTGGCGCCGCAGTAACAGCGTTAACATTGCCATC
GCGCCGTTCATCTATTACAATAAGGTTTTAGCTCAATTGTCAACTAGGTTTAAGATTTCG
CAATAAAACTACAATGGGTACAAAACAAAATTTGTTTTATTTCATTTTCTCAAATACACG
ATTCGATCCACCAGGGTTTCCTCGGTATATTCCTAGAAGTCCACATGCGATCCGCACATG
ATGTAGGTCGCATCTCGTCGAACCGTTGCGCCGACAATCTATTCATCTGTACGTGCATCA
ATGTGATGAAGTCATCGAGACACTGTGCAAAATCAACGGGGCAGCTGCCGACGATGCGGT
CCGTCAACGTGCCGACAATCGTGCACTTGTGACGCTCAAGTCATCGACGTAGTGTCTAAC
GGTAGCGGCCAGCAGTTCACGATCTCGTCCGAGTGTGGGCGTGTTGATACCGTGGTCGGT
CTCAGCGCGTACACGAGCGGTGTGATACGACGATTGTCGTTCCTGATCGTGCCGTTCAGG
ATTCTCGAGTGAGTCTTGTGTATTTCATGGTAGTTACGTAGATATGCACCCTCGGTGGGA
TCGATGACGCTAGTCAAACGCATGGTGGATGGGTAGAGACACGGCCACCGAATGGTGATC
ACGCACACCGCCTATGGTTGCATTATTCCGTAAGTGCCGGGTACTCGTGCCATATCAACT
CGGCACACACCCACACCGACAGATGTTTCGAAGTCGCTACTGCGCAAGCCTTCTAGCGCA
GCGTGTAAAATAGTTATGGAATTGGAATGTAATGCGTTCACGGGCCGCTGTTGCTCGATC
AAGTCGATCAGCTCGTCCACCAGTTGCAGTCGATATCTTCTCGGGTCCGATGTCTGTAGC
CAAGTCGATGAAATCGTGCGTGGTACGTGCGGAAATGCTTCCCCACTCCAATCTATGGTG
CCGTATTCGAAAACACACCTACACTTGCCGATACAC

Tet-108 (50.1% GC)
ATGAAATCTAACAATGCGCTCATCGTCATCCTCGGCACCGTCACCCTGGATGCTGTAGGC
ATAGGCTTGGTTATGCCGGTACTGCCGGGCCTCTTGCGGGATATCGTCCATTCCGACAGC
ATCGCCAGTCACTATGGCGTGCTGCTAGCGCTATATGCGTTGATGCAATTTCTATGCGCA
CCCGTTCTCGGAGCACTGTCCGACCGCTTTGGCCGCCGCCCAGTCCTGCTCGCTTCGCTA
CTTGGAGCCACTATCGACTACGCGATCATGGCGACCACACCCGTCCTGTGGATCCTCTAC
GCCGGACGCATCGTGGCCGGCATCACCGGCGCCACAGGTGCGGTTGCTGGCGCCTATATC
GCCGACATCACCGATGGGGAAGATCGGGCTCGCCACTTCGGGCTCATGAGCGCTTGTTTC
GGCGTGGGTATGGTGGCAGGCCCCGTGGCCGGGGGACTGTTGGGCGCCATCTCCTTGCAT
GCACCATTCCTTGCGGCGGCGGTGCTCAACGGCCTCAACCTACTACTGGGCTGCTTCCTA
ATGCAGGAGTCGCATAAGGGAGAGCGTCGACCGATGCCCTTGAGAGCCTTCAACCCAGTC
AGCTCCTTCCGGTGGGCGCGGGGCATGACTATCGTCGCCGCACTTATGACTGTCTTCTTT
ATCATGCAACTCGTAGGACAGGTGCCGGCAGCGCTCTGGGTCATTTTCGGCGAGGACCGC
TTTCGCTGGAGCGCGACGATGATCGGCCTGTCGCTTGCGGTATTCGGAATCTTGCACGCC
CTCGCTCAAGCCTTCGTCACTGGTCCCGCCACCAAACGTTTCGGCGAGAAGCAGGCCATT
ATCGCCGGCATGGCGGCCGACGCGCTGGGCTACGTCTTGCTGGCGTTCGCGACGCGAGGC
TGGATGGCCTTCCCCATTATGATTCTTCTCGCTTCCGGCGGCATCGGGATGCCCGCGTTG
CAGGCCATGCTGTCCAGGCAGGTAGATGACGACCATCAGGGACAGCTTCAAGGATCGCTC
GCGGCTCTTACCAGCCTAACTTCGATCACTGGACCGCTGATCGTCACGGCGATTTATGCC
GCCTCGGCGAGCACATGGAACGGGTTGGCATGGATTGTAGGCGCCGCCCTATACCTTGTC
TGCCTCCCCGCGTTGCGTCGCGGTGCATGGAGCCGGGCCACCTCGACCTGAATGGAAGCC
GGCGGCACCTCGCTAA
TGGAGCGTTACCGTTTATGCCAATCGCAATGTGCACAACATACATGGGAAGAAAAAGTAT
TCTTGAAGTTGTAGAACACGTGAAAGATTATGGAGGAACAGTGGTGTACGGGGACACGGA
CTGCCTTTGTCGAACCACTCCTGTTTTAATTCGAAAAGACGACAGCGATGTTTTTTTAC

AACTGTGGATGAGTTAAGTAAAAATGACTGGGAGTACATAAATCCCAACAAAGACATCTC
GTCCCCCAAAGAAAATTACGAAATATGGAGTGACACAGGATTTACAAAGATCGAAAATGT
TGTAAGATGTAAAATAGACGAGCCCCTAAAACGTGTAACTACACATGTGGGCTCTGTAAT
TTGTTCCGACAACCACTCCTTACTCACCGAAAATTTGACAGGTGTAACACCAAATGATGT
TGTTATAGGGGATAGATTGTGTGTAAGAGAACTTCCGTTACAACAAGATACACCACAAGT
TCCAATATATATAAATAGACTTACCGCAGAGAGGATCCAAATGTATGAAATCCCAGAAGT
TTCTTACCTAAACGTGTCTGCCAAAATGGCTTTTGTATGGGGATTATTTTTTGCGGACGG
TGGTGCCGGTATTTACAAACAAAGCAAATCTAATTGTTTGTCTAATTTCACATGGGCTAT
CAATAAAGCTCTGTCACGTTATGAAATGGGAGGATCATGTGTCATGTGAACATGATCCAA
AAATCATCAGAAAAAAAGAATTAACAGTCAAAATAGACGAATTGAAAAGAAAGATTAAGG
ATGAAAAGGACAGGGGTAGGAAGGATAAGTTAAAAATAGACTTGGCGTCCTTAACAAAAG
AAAGGTCTAACATAACAAAAAAACTTAGTAAAAGTGTTATGTGTGCTAAAAGAAGTTATA
GATTTTTAAAAGAGCCTCGAGGAGTTCTTCCGACAATCATAAAGGATCTTCTGGACGCCA
GAAAAAACACTCGTGCGGAAATAAAAATATGGCAAAATAAACTTAAATCAGCAGGTGGTC
TTGAAGAACAAGAAATCGCAAAATCAATGATCCAAATTCTGGACCAAAGACAAAATTCGT
ACAAAGTCAGTGCTAACTCAATGTATGGAGCAACCGGTGTTAG

Tet-116 (53.2% GC)

ATGAAATCTAACAATGCGCTCATCGTCATCCTCGGCACCGTCACCCTGGATGCTGTAGGC
ATAGGCTTGGTTATGCCGGTACTGCCGGGCCTCTTGCGGGATATCGTCCATTCCGACAGC
ATCGCCAGTCACTATGGCGTGCTGCTAGCGCTATATGCGTTGATGCAATTTCTATGCGCA
CCCGTTCTCGGAGCACTGTCCGACCGCTTTGGCCGCCGCCCAGTCCTGCTCGCTTCGCTA
CTTGGAGCCACTATCGACTACGCGATCATGGCGACCACACCCGTCCTGTGGATCCTCTAC
GCCGGACGCATCGTGGCCGGCATCACCGGCGCCACAGGTGCGGTTGCTGGCGCCTATATC
GCCGACATCACCGATGGGGAAGATCGGGCTCGCCACTTCGGGCTCATGAGCGCTTGTTTC
GGCGTGGGTATGGTGGCAGGCCCCGTGGCCGGGGGACTGTTGGGCGCCATCTCCTTGCAT
GCACCATTCCTTGCGGCGGCGGTGCTCAACGGCCTCAACCTACTACTGGGCTGCTTCCTA
ATGCAGGAGTCGCATAAGGGAGAGCGTCGACCGATGCCCTTGAGAGCCTTCAACCCAGTC
AGCTCCTTCCGGTGGGCGCGGGGCATGACTATCGTCGCCGCACTTATGACTGTCTTCTTT
ATCATGCAACTCGTAGGACAGGTGCCGGCAGCGCTCTGGGTCATTTTCGGCGAGGACCGC
TTTCGCTGGAGCGCGACGATGATCGGCCTGTCGCTTGCGGTATTCGGAATCTTGCACGCC
CTCGCTCAAGCCTTCGTCACTGGTCCCGCCACCAAACGTTTCGGCGAGAAGCAGGCCATT
ATCGCCGGCATGGCGGCCGACGCGCTGGGCTACGTCTTGCTGGCGTTCGCGACGCGAGGC
TGGATGGCCTTCCCCATTATGATTCTTCTCGCTTCCGGCGGCATCGGGATGCCCGCGTTG
CAGGCCATGCTGTCCAGGCAGGTAGATGACGACCATCAGGGACAGCTTCAAGGATCGCTC
GCGGCTCTTACCAGCCTAACTTCGATCACTGGACCGCTGATCGTCACGGCGATTTATGCC
GCCTCGGCGAGCACATGGAACGGGTTGGCATGGATTGTAGGCGCCGCCCTATACCTTGTC
TGCCTCCCCGCGTTGCGTCGCGGTGCATGGAGCCGGGCCACCTCGACCTGAATGGAAGCC
GGCGGCACCTCGCTAA
GCGGAGATTTTATAAGCGAGCTGTGACTTATCGAGTATATCTCGAGCWGAGGATCGTAAC
TTCTTCATTTCAGCTCGTACCTTCCTGCGAGCGTCTAAGAAGTATTCAACCAATCTGGTA
GTGTCTCATGCGAGTTTTCAGTATACGAACTCGTTGTTTTGCGCACAGTCTAGAACCACC
AGACATTCTTGACTTTAGCACTTTCAATCGGATGGCGGCCCGCTGTAAATCAACCGACGA
CAGCTTGATTGATGGTATCGGAGAACCGTTGTAATTATCATCGGTAAAACCATTACATTC
CGAGTAATCTTCAACGTCGTCAACTGGCTTACGCGTTGTACTTGTCGGCTTGAAGTACTT
TGTAATCGGCTGAACCCCGATGCATTTTCGCTTCTCGACTTTTGCTAGATCTTCGAATAT
TGACTTTAGTTTTTCGCGCTCTTTTATGTTTGGATCGTGCTCGCAACCAACGTGATCCTC
CCATTCTACAATTTCAAAGTTTTCGTCGGTGTAGTCACTGTGTGCATCGTCGTCGATGAC
CGTACTGTAGCAGAGATTATAAGCCATCATCAACGTCGGATATATCCGACGTTGATGATG
GTTATAATCTCTGCTACAGTACGGTCATCGACGACGATGCACACAGTGACTACACCGACG
AAAACTTTGAAATTGTAGAATGGGAGGATCACGTTGGTTGCGAGCACGATCCAAACATAA
AAGAGCGCGAAAAACTAAAGTCAATATTCGAAGATCTAGCAAAAGTCGAGAAGCGAAAAT
GCATCGGGGTTCAGCCGATTACAAAGTACTTCAAGCCGACAAGTACAACGCGTAAGCAGT
TGACGACGTTGAAGATTACTCGGAATGTAATGTTTTACCGATGATAATTACAACGGTTCT

CCGATACCATCAATCAAGCTGTCGTCGGTTGATTTACAGCGGGCCGCCATCCGATTGAAA
GTGCTAAAGTCAAGAATGTCTGGTGGTTCTAGACTGTGCGCAAAACAACGAGTTCGTATA
CTGAAAACTCGCAGAGGACTACTACCAGATTTGGTTGAATACTTCTTAGACGCTCGCAGG
AAGGTACGAGCTGAAATGAAGAACGTTAGCGATCCTCTAGCTCGAGATATACTCGATAAG
T

Tet-193 (59.1% GC)

ATGAAATCTAACAATGCGCTCATCGTCATCCTCGGCACCGTCACCCTGGATGCTGTAGGC
ATAGGCTTGGTTATGCCGGTACTGCCGGGCCTCTTGCGGGATATCGTCCATTCCGACAGC
ATCGCCAGTCACTATGGCGTGCTGCTAGCGCTATATGCGTTGATGCAATTTCTATGCGCA
CCCGTTCTCGGAGCACTGTCCGACCGCTTTGGCCGCCGCCCAGTCCTGCTCGCTTCGCTA
CTTGGAGCCACTATCGACTACGCGATCATGGCGACCACACCCGTCCTGTGGATCCTCTAC
GCCGGACGCATCGTGGCCGGCATCACCGGCGCCACAGGTGCGGTTGCTGGCGCCTATATC
GCCGACATCACCGATGGGGAAGATCGGGCTCGCCACTTCGGGCTCATGAGCGCTTGTTTC
GGCGTGGGTATGGTGGCAGGCCCCGTGGCCGGGGGACTGTTGGGCGCCATCTCCTTGCAT
GCACCATTCCTTGCGGCGGCGGTGCTCAACGGCCTCAACCTACTACTGGGCTGCTTCCTA
ATGCAGGAGTCGCATAAGGGAGAGCGTCGACCGATGCCCTTGAGAGCCTTCAACCCAGTC
AGCTCCTTCCGGTGGGCGCGGGGCATGACTATCGTCGCCGCACTTATGACTGTCTTCTTT
ATCATGCAACTCGTAGGACAGGTGCCGGCAGCGCTCTGGGTCATTTTCGGCGAGGACCGC
TTTCGCTGGAGCGCGACGATGATCGGCCTGTCGCTTGCGGTATTCGGAATCTTGCACGCC
CTCGCTCAAGCCTTCGTCACTGGTCCCGCCACCAAACGTTTCGGCGAGAAGCAGGCCATT
ATCGCCGGCATGGCGGCCGACGCGCTGGGCTACGTCTTGCTGGCGTTCGCGACGCGAGGC
TGGATGGCCTTCCCCATTATGATTCTTCTCGCTTCCGGCGGCATCGGGATGCCCGCGTTG
CAGGCCATGCTGTCCAGGCAGGTAGATGACGACCATCAGGGACAGCTTCAAGGATCGCTC
GCGGCTCTTACCAGCCTAACTTCGATCACTGGACCGCTGATCGTCACGGCGATTTATGCC
GCCTCGGCGAGCACATGGAACGGGTTGGCATGGATTGTAGGCGCCGCCCTATACCTTGTC
TGCCTCCCCGCGTTGCGTCGCGGTGCATGGAGCCGGGCCACCTCGACCTGAATGGAAGCC
GGCGGCACCTCGCTAA
ACATCGAACACGCCAAGGCGATCGCCGATGAACAAGCGGCTCGTGCCCTTGTACGTTGCG
ATGTACAGTAACTGGTCGAATACAAACGCACCGACTCGAGCCACCATGGACATCGAAGCG
ATACGCGGACGTTACACCGTCTCCGTACCGGAGAGCATGGAACGTCCGATGGTGATTCTG
TTCAACGACTTGACAATCTCCGAGGATTTGGTGGTGTTCGCGACGCACGGTGAGTTGATC
AAGTACGACACTCGATACCCGCCGTCGTCGGACGCAGCGCTGGCCGCCATGAGCAGTCGT
CGCGCCAGGAAGGGTGGCGAACGAGACAAACGCATCTCGTTGTACATGGTGCGACGCGGT
ATCTACTCGCCGGGCATGAACTACGACATAGCGCACGTACACCAGACTCCCGGCGTGGGC
TACGTGATCACGGTGATCAGTAATAACGCGCCCACCGTCGACGTGCCTCGAAGTCTAGTC
ACCGCACTCGGCATGGGCGGTGACGACGATCAGGTGGTGGTGAGCGATGTCGGCGGCGGT
GTTACATACACCGCGTCGTTCGTCCTAAACGACATCACGTTCGTCACAGTACCTGCAACA
TTACACGTTGCTCAAGTGTACCGACAAGGCGACGTGTATATTCGTCGACGAAACGCATCT
GATGAAGTACGCGCGCCCTTGGCGTCGATACACAATCGCCAGTCCGCGATTCATATACGA
GTACAATAATTTCACGGTGAAGTTTAGCGCGACCGTGGAGAGCACGCGCAGGGACGTGGC
TCGAGTGTCGCCCTATTGCCGTATCCGTATCTACGATGCGCCCAACACGCAGATCATGTA
CGGCATGGCGAACGACATAACCGCACACATGATCGAGTACAAGAGTAGGGAGCGTTCGAT
CCCGTCTCTGTACGCCGAGAATCTACACAACGATCAGCTGTTGCGGATCAACGTGCGCAT
AATGCGTGACTCGAAACCGTTAAACGCGAACCCGGCTACCTGGCTCGGCAACACCGACAG
GTCCGTAGAAAACTGCAAGCGCAAAAGACCGGCACCAACGCAGACGTACGCTCCCTGATC
GGTGTCAACAACTACGCCAGACAATGTGCACGCCTACCTACAGCCGT

Tet-240 (54.5% GC)

ATGAAATCTAACAATGCGCTCATCGTCATCCTCGGCACCGTCACCCTGGATGCTGTAGGC
ATAGGCTTGGTTATGCCGGTACTGCCGGGCCTCTTGCGGGATATCGTCCATTCCGACAGC
ATCGCCAGTCACTATGGCGTGCTGCTAGCGCTATATGCGTTGATGCAATTTCTATGCGCA
CCCGTTCTCGGAGCACTGTCCGACCGCTTTGGCCGCCGCCCAGTCCTGCTCGCTTCGCTA
CTTGGAGCCACTATCGACTACGCGATCATGGCGACCACACCCGTCCTGTGGATCCTCTAC

GCCGGACGCATCGTGGCCGGCATCACCGGCGCCACAGGTGCGGTTGCTGGCGCCTATATC
GCCGACATCACCGATGGGGAAGATCGGGCTCGCCACTTCGGGCTCATGAGCGCTTGTTTC
GGCGTGGGTATGGTGGCAGGCCCCGTGGCCGGGGGACTGTTGGGCGCCATCTCCTTGCAT
GCACCATTCCTTGCGGCGGCGGTGCTCAACGGCCTCAACCTACTACTGGGCTGCTTCCTA
ATGCAGGAGTCGCATAAGGGAGAGCGTCGACCGATGCCCTTGAGAGCCTTCAACCCAGTC
AGCTCCTTCCGGTGGGCGCGGGGCATGACTATCGTCGCCGCACTTATGACTGTCTTCTTT
ATCATGCAACTCGTAGGACAGGTGCCGGCAGCGCTCTGGGTCATTTTCGGCGAGGACCGC
TTTCGCTGGAGCGCGACGATGATCGGCCTGTCGCTTGCGGTATTCGGAATCTTGCACGCC
CTCGCTCAAGCCTTCGTCACTGGTCCCGCCACCAAACGTTTCGGCGAGAAGCAGGCCATT
ATCGCCGGCATGGCGGCCGACGCGCTGGGCTACGTCTTGCTGGCGTTCGCGACGCGAGGC
TGGATGGCCTTCCCCATTATGATTCTTCTCGCTTCCGGCGGCATCGGGATGCCCGCGTTG
CAGGCCATGCTGTCCAGGCAGGTAGATGACGACCATCAGGGACAGCTTCAAGGATCGCTC
GCGGCTCTTACCAGCCTAACTTCGATCACTGGACCGCTGATCGTCACGGCGATTTATGCC
GCCTCGGCGAGCACATGGAACGGGTTGGCATGGATTGTAGGCGCCGCCCTATACCTTGTC
TGCCTCCCCGCGTTGCGTCGCGGTGCATGGAGCCGGGCCACCTCGACCTGAATGGAAGCC
GGCGGCACCTCGCTAA
TCGCCGTTGCTGTCGCCTACTGTCGGCGCCTTGTTGATGCTGTTGTTTCTCGATCGAGTG
GTTGGTGAGTGCGTTATCGGCAATTTCGCTGAAACTTTTCAAATCTTCCACACCCTCCGT
CAACGTTCTCCACGTACCGCGTTGGCCGTTGAATCGTCGGAACAGAGCCAGGGCGGCGAA
CAATGCCGCGATCGCTATGATCGTGTACTTGTTGCTGAACACCGATTCGGCGTCGTTCAT
AGCCCTCTTCCAAGCCAACCAAGCACCCATCAATGCTACGGACACGATAAGCACATGCTT
AATTTCCATTTACCCAGTCGAAACAGCGAAGGACGGTAACGGATACAAAGAAAATAAAAG
ACACATTTTTAGATTAACCATTTATTGACACACAGTGTTACCACAAAGATCGCAATAACC
GTCGACGTCACCATCACGTTTGGGACAGTACTGTAGGAATCGCACATCGACCGCTATACT
GTTCGTACAGTACAGGAAGGCAAACGTAAAGTCCACAAACGACCTCGGAGAGTTGACTGC
AAAAAATAACACCATCTGGTTCCGTAAATGCCAGAGAGATTCACTCGCCGCTCCGGTTAT
GTCGCGCAATTGCACCAGGTTGGATCGTGGAAATTGTAAGTATGTTTTGTTGAAACGTTT
CATTATCACCGTCGTATAGTTACCACGGTGCACTCGGATCTTTGCACTGACCACTCGACG
AATCACACCGGTGCGTGATCTTCGCAACAGACAATATTCATCACCCGTACTGGGATCTAT
GAAGACGTCGAATATTGTCGAGTAAAAGTTATGCGTGTCGTATGCACAACCCAGCGAAGG
TCTGGAGATAATCGAACGCATCATCATCTTCTCACCACCACAATGACCGATGAACGAATA
ATACCAATCGTCGTACTGCATCTCGCGATTAACTGTCCTCGCCCGTTTAATACATTTCGA
TTTATATGTTTTTATGCTGTAACACTAATGTGTATGTTACACGCGAACAGAAACGTGACT
TCTTGAACTGCGACCGGTCGGTCGAGCGAGCGATCAATCGTAACGTTGACTGTATTGTAT
TATTGTCGAAACTACACTGTCCTCGAATGTTTTGTATCTGTCCACCGTCGACGA

Tet-289 (53.3% GC)
ATGAAATCTAACAATGCGCTCATCGTCATCCTCGGCACCGTCACCCTGGATGCTGTAGGC
ATAGGCTTGGTTATGCCGGTACTGCCGGGCCTCTTGCGGGATATCGTCCATTCCGACAGC
ATCGCCAGTCACTATGGCGTGCTGCTAGCGCTATATGCGTTGATGCAATTTCTATGCGCA
CCCGTTCTCGGAGCACTGTCCGACCGCTTTGGCCGCCGCCCAGTCCTGCTCGCTTCGCTA
CTTGGAGCCACTATCGACTACGCGATCATGGCGACCACACCCGTCCTGTGGATCCTCTAC
GCCGGACGCATCGTGGCCGGCATCACCGGCGCCACAGGTGCGGTTGCTGGCGCCTATATC
GCCGACATCACCGATGGGGAAGATCGGGCTCGCCACTTCGGGCTCATGAGCGCTTGTTTC
GGCGTGGGTATGGTGGCAGGCCCCGTGGCCGGGGGACTGTTGGGCGCCATCTCCTTGCAT
GCACCATTCCTTGCGGCGGCGGTGCTCAACGGCCTCAACCTACTACTGGGCTGCTTCCTA
ATGCAGGAGTCGCATAAGGGAGAGCGTCGACCGATGCCCTTGAGAGCCTTCAACCCAGTC
AGCTCCTTCCGGTGGGCGCGGGGCATGACTATCGTCGCCGCACTTATGACTGTCTTCTTT
ATCATGCAACTCGTAGGACAGGTGCCGGCAGCGCTCTGGGTCATTTTCGGCGAGGACCGC
TTTCGCTGGAGCGCGACGATGATCGGCCTGTCGCTTGCGGTATTCGGAATCTTGCACGCC
CTCGCTCAAGCCTTCGTCACTGGTCCCGCCACCAAACGTTTCGGCGAGAAGCAGGCCATT
ATCGCCGGCATGGCGGCCGACGCGCTGGGCTACGTCTTGCTGGCGTTCGCGACGCGAGGC
TGGATGGCCTTCCCCATTATGATTCTTCTCGCTTCCGGCGGCATCGGGATGCCCGCGTTG
CAGGCCATGCTGTCCAGGCAGGTAGATGACGACCATCAGGGACAGCTTCAAGGATCGCTC

GCGGCTCTTACCAGCCTAACTTCGATCACTGGACCGCTGATCGTCACGGCGATTTATGCC
GCCTCGGCGAGCACATGGAACGGGTTGGCATGGATTGTAGGCGCCGCCCTATACCTTGTC
TGCCTCCCCGCGTTGCGTCGCGGTGCATGGAGCCGGGCCACCTCGACCTGAATGGAAGCC
GGCGGCACCTCGCTAA
TACGATCCCGGCGTAGTCTCCGATAACTCCGTACACGAATTGTCGCACAATCTTGGTACA
ATGCGGCGAACCCATGAGCGAACACCATAAAGCGCCCACGAAAGATTCCATCACGTCCTC
GAGTAACGATATCACGTCTCGATGTATGTGGGTGGAGGTCGTGTCTGTGTACACGGAATC
GGCACACACTTTCGGTTGACATTGAACGCCGGACGCGTTGTTGCGTTTGAATCTGTGTCC
GTACACTTGTAATTTTCTACCATCGACACCGGCGTACTGGCACCTTATGAACTTGTTCAA
TCCGATCGATACACAACACTTTGACAGATTCCACTTTCCCACGTAGATGCTGCGTAGTTT
CGTCATCACACCGATGTCTTGCTGAATGTTATGTATATCCGTGTAGTTTTCACGAATGTA
GTCTATTATGAAGCGGTTGATGACGGAATCACCGATGTACTCGAAGTATTCGTAGTTGAA
TATGTTGTCGTATTCTTTGGAGGTGAGCGCCGCTTGAAATTTATCCATATTACCCTCATC
ACGAAACGTGTCCAATAAATGTTTATAGTATTGCGCGTCGACGTTACCATATTCGATGAC
AATTTCGGCAATGTGTTCGGCGGTTATGATGACGGGGGTGGCACATCCGTTACCGTCTCC
TTGTGGTGACATATCGATTAAGTTGCCTTCATCCAGCGCCCAATGTAAAACCCTCGCTCT
CCAATGTCGACCGCTGTACTCGGCCGTGTAACGGTTGTGTGTAATGCAACGTTTCGGAAC
TAAGACAAATTTAGCGGTGGTGGTGGTGGTGGTTGTTCTTTTGAACGATGACCTTCTCTT
TTTGAAATGGTCGATTTTATTGAACGGTTTTTCCTTGACGTTCCGGTTGGCAGTTCTATA
ACGCAAGAGAAACGACTTATCAACGTGGTTATACTTTGACGTCCGTGAAACGACTGTGGT
GTTAGCATCAATTGTTGTTGTTGTACGGTTGTTGTCGTACGCTTGTTGTTGTGTTGTAAC
ATCGGTCTCATTGTTACAATTCATACTGAACGATATGTGATTTAATTAATACACACAAAC
ACGTGTCATGTGTACACGGATGTTCCCATATACTGATGATGATATGTGGAAGGGGGAAT
TGAAACAACTCATTCGT

Tet-523 (54.9% GC)

ATGAAATCTAACAATGCGCTCATCGTCATCCTCGGCACCGTCACCCTGGATGCTGTAGGC
ATAGGCTTGGTTATGCCGGTACTGCCGGGCCTCTTGCGGGATATCGTCCATTCCGACAGC
ATCGCCAGTCACTATGGCGTGCTGCTAGCGCTATATGCGTTGATGCAATTTCTATGCGCA
CCCGTTCTCGGAGCACTGTCCGACCGCTTTGGCCGCCGCCCAGTCCTGCTCGCTTCGCTA
CTTGGAGCCACTATCGACTACGCGATCATGGCGACCACACCCGTCCTGTGGATCCTCTAC
GCCGGACGCATCGTGGCCGGCATCACCGGCGCCACAGGTGCGGTTGCTGGCGCCTATATC
GCCGACATCACCGATGGGGAAGATCGGGCTCGCCACTTCGGGCTCATGAGCGCTTGTTTC
GGCGTGGGTATGGTGGCAGGCCCCGTGGCCGGGGGACTGTTGGGCGCCATCTCCTTGCAT
GCACCATTCCTTGCGGCGGCGGTGCTCAACGGCCTCAACCTACTACTGGGCTGCTTCCTA
ATGCAGGAGTCGCATAAGGGAGAGCGTCGACCGATGCCCTTGAGAGCCTTCAACCCAGTC
AGCTCCTTCCGGTGGGCGCGGGGCATGACTATCGTCGCCGCACTTATGACTGTCTTCTTT
ATCATGCAACTCGTAGGACAGGTGCCGGCAGCGCTCTGGGTCATTTTCGGCGAGGACCGC
TTTCGCTGGAGCGCGACGATGATCGGCCTGTCGCTTGCGGTATTCGGAATCTTGCACGCC
CTCGCTCAAGCCTTCGTCACTGGTCCCGCCACCAAACGTTTCGGCGAGAAGCAGGCCATT
ATCGCCGGCATGGCGGCCGACGCGCTGGGCTACGTCTTGCTGGCGTTCGCGACGCGAGGC
TGGATGGCCTTCCCCATTATGATTCTTCTCGCTTCCGGCGGCATCGGGATGCCCGCGTTG
CAGGCCATGCTGTCCAGGCAGGTAGATGACGACCATCAGGGACAGCTTCAAGGATCGCTC
GCGGCTCTTACCAGCCTAACTTCGATCACTGGACCGCTGATCGTCACGGCGATTTATGCC
GCCTCGGCGAGCACATGGAACGGGTTGGCATGGATTGTAGGCGCCGCCCTATACCTTGTC
TGCCTCCCCGCGTTGCGTCGCGGTGCATGGAGCCGGGCCACCTCGACCTGAATGGAAGCC
GGCGGCACCTCGCTAA
CGACGACTCCCGGTAATGTGTCACCACCGTTGTTAACGACACCGACCTCGTTGGGTAGGC
GCGGATAACTGATCGCTGTGCTTCCACTCATTTACCATTGTGAGATGAAAATGGTTCAAG
CACAGTGTTCAGCTACAAAGCTATCAATGCTTATCGTATGCATCATCATCATGATTACAA
CCACGCTACCGGATCACGGTTGTATGGGTTTCGACGTTAAAAGCACAATAGTAGCCGCTT
TAGATCCGGTGACTTCGTTGATTAACCGCGTCAAATCGTCAATCGAAGACGTCAAGACCG
AGGTTAACACCGTTATAGAGGACGTTAAATCGTTGAAGAACGACGCCATCAACAAAGTCA

GCGAAGTGGTATCAACGACTAAATCGATGGCGGTGGAAATTAAACAATTCACTAATTTAA
CAACATTTATTCATAAACTTAGGAATGTAATAACAAAACAAGACATCTTAGACCTAGAGA
CGGGCGTTATAAATTTCATACTAGACTTGTCCGTACACCTGGCGGTTACCGTTCATAAAC
GTTGGCCGATGTTAGTGTACGCGGCGGCCGCATGTACCGCCCTTGTAGCTACCATAATCA
TCATCTTGACTATATCGATATGCGTGCTGTCGTGTCGACTACGAAGATTCGACGATGCCA
TCAAGGACACGGTTGAATGATGCGAGTACGTCTTCGCGTGGCAAGTTTCCGTTCAACACG
AAAACTGAACTATGGTTGTCGACGTATCGTTTCGTGTAGCATTCGACTCTTTCGAAATAC
TCGACGTCCAATGATTTCTCCGCCTCGCGTCCTCGTTCCTGAGCTCGGCGGTGACAGGTG
TCCGGTTTCGTGTCAATGTACACGACGTGGTCGATTCTCGGAATGTCATTCTCCAAACTG
CGAAACACATCCGTGTACACGTCGAATTGTTTGTCGGTGATCGAGCCTCGGTCTCGCATC
ATTCTGGTGAAGACGTCGTACGCCGACCACGGCGAGCGTTCCATGATAACGACTCGAGCT
CCGGTTCGCAGACATGTCTCGTACGAGTCCAACCACTTTTGATATTTACTCGTCAGCACT
TGCAGCTCGAAGGGGAAAGCGTATC
